# Mtf2-PRC2 control of canonical Wnt signaling is required for definitive erythropoiesis

**DOI:** 10.1038/s41421-018-0022-5

**Published:** 2018-05-01

**Authors:** Janet L. Manias Rothberg, Harinad B. Maganti, Hani Jrade, Christopher J. Porter, Gareth A. Palidwor, Christopher Cafariello, Hannah L. Battaion, Safwat T. Khan, Theodore J. Perkins, Robert F. Paulson, Caryn Y. Ito, William L. Stanford

**Affiliations:** 1 The Sprott Center for Stem Cell Research, Regenerative Medicine Program, Ottawa Hospital Research Institute, Ottawa, ON K1H 8L6 Canada; 2 Ottawa Institute of Systems Biology, Ottawa, ON Canada; 3 Department of Cellular and Molecular Medicine, University of Ottawa, Ottawa, ON Canada; 4 Department of Biochemistry, Microbiology and Immunology, University of Ottawa, Ottawa, ON Canada; 5 Ottawa Bioinformatics Core Facility, The Sprott Center for Stem Cell Research, Ottawa Hospital Research Institute, Ottawa, ON K1H 8L6 Canada; 6 Department of Veterinary and Biomedical Sciences, Pennsylvania State University, University Park, PA 16802 USA

## Abstract

Polycomb repressive complex 2 (PRC2) accessory proteins play substoichiometric, tissue-specific roles to recruit PRC2 to specific genomic loci or increase enzymatic activity, while PRC2 core proteins are required for complex stability and global levels of trimethylation of histone 3 at lysine 27 (H3K27me3). Here, we demonstrate a role for the classical PRC2 accessory protein Mtf2/Pcl2 in the hematopoietic system that is more akin to that of a core PRC2 protein. *Mtf2*^*−/−*^ erythroid progenitors demonstrate markedly decreased core PRC2 protein levels and a global loss of H3K27me3 at promoter-proximal regions. The resulting de-repression of transcriptional and signaling networks blocks definitive erythroid development, culminating in *Mtf2*^*−/−*^ embryos dying by e15.5 due to severe anemia. Gene regulatory network (GRN) analysis demonstrated Mtf2 directly regulates Wnt signaling in erythroblasts, leading to activated canonical Wnt signaling in Mtf2-deficient erythroblasts, while chemical inhibition of canonical Wnt signaling rescued Mtf2-deficient erythroblast differentiation in vitro. Using a combination of in vitro, in vivo and systems analyses, we demonstrate that Mtf2 is a critical epigenetic regulator of Wnt signaling during erythropoiesis and recast the role of polycomb accessory proteins in a tissue-specific context.

## Introduction

Epigenetic regulation of cell signaling is fundamental to developmental and homeostatic processes. The evolutionarily conserved polycomb group proteins were first identified by their repression of *Homeotic* (*Hox*) genes regulating body axis formation. The different polycomb group proteins associate to form functionally distinct complexes that belong to two major families: polycomb repressive complexes 1 and 2 (PRC1 and PRC2, respectively). While the core components of the PRC1 include Pcgf and an E3 ubiquitin ligase Ring1b, responsible for the maintenance of H2AK119ub1 marks^1^, PRC2 consists of three core proteins: Suz12, Eed and one of the histone methyltransferases Ezh1 or Ezh2, responsible for the maintenance of the global H3K27me3 marks^2,3^. Although the core PRC2 proteins are required for the stability of the PRC2 complex, none encode DNA binding domains. Hence, the core PRC2 proteins interact with accessory proteins, such as Jarid2, Aebp2 or members of the *polycomb-like* (*Pcl*) family encoding Phf1 (Pcl1), Mtf2/Pcl2 and Phf19 (Pcl3) that contain DNA binding domains able to target the PRC2 to specific genomic loci^4–7^. While the core PRC2 complex proteins are abundantly expressed across all tissues and are required for proper differentiation of embryonic stem cells (ESCs) and somatic stem cells^8,9^, PRC2 accessory proteins have shown limited expression in certain tissues and hence need to be studied in a tissue-specific manner.

Hematopoiesis is a tightly regulated process requiring the lifelong generation of sufficient mature cells of multiple lineages to replace aged cells. During hematopoiesis, polycomb proteins play fundamental roles in cell fate decisions and differentiation programs. Core components of PRC2 have significant roles in regulating hematopoiesis and recent studies have demonstrated that Ezh1 and Ezh2 are differentially regulated during hematopoietic ontogeny^10^. Conditional ablation of Ezh2 and Eed in hematopoietic cells revealed defects in hematopoietic stem cell (HSC) self-renewal and erythroid differentiation, ultimately leading to anemia^10,11^. However, the role of Ezh2 is cell-stage specific, as Ezh2 is dispensable in adult HSCs via compensatory Ezh1 function^10^. Ablation of the PRC2 accessory protein Jarid2 resulted in anemia via non-cell-autonomous mechanisms^12,13^. While extensive studies have been conducted on PRC2 core complex members and Jarid2 in the hematopoietic system, little is known about the role of other PRC2 accessory proteins within this context.

Metal regulatory transcription factor 2 (Mtf2; a.k.a., *polycomb-like 2* (*Pcl2*)) is a catalytically inactive Pcl family protein that has been shown to recruit the PRC2 to target gene loci within ESCs^14,15^. Mtf2 knockdown in ESCs led to reduced H3K27me3 levels at Mtf2 target loci but did not affect global H3K27me3 levels^14,15^. Moreover, manipulating the expression of Mtf2 did not affect the expression of core PRC2 complex members^15,16^, supporting its role as a PRC2 accessory protein in ESCs. While the function of Mtf2 has been characterized in ESCs, the role of Mtf2 in vivo is poorly understood.

The Wnt/β-catenin signaling pathway is highly conserved for body axis formation, endodermal specification and organ formation among vertebrates^17–19^. Abnormal expression of Wnt/β-catenin signaling results in developmental disorders and diseases including cardiovascular diseases, skeletal disorders, neuronal diseases and cancer^20–22^. A variety of transcriptional and post-transcriptional mechanisms impart tight regulation on the Wnt/β-catenin signaling pathway^21^. Recently, PRC2-mediated regulation of Wnt signaling has been shown to control cell growth across multiple tissues^23,24^; however, it is not clear how the Wnt signaling pathway is epigenetically regulated and little is known about the role of PRC2 in regulating these pathways.

Here we show the PRC2 accessory protein, Mtf2, is required for embryonic and erythroid development by epigenetically repressing Wnt transcriptional and signaling pathways during hematopoiesis. In CD71^+^Ter119^+^ erythroblasts, Mtf2 deficiency results in a global loss of promoter-proximal H3K27me3, aligning the function of Mtf2 in erythropoiesis more similar to a core PRC2 protein. We further demonstrate that Mtf2-PRC2 represses canonical Wnt signaling, which regulates erythroid maturation by controlling critical differentiation genes, including *Gata2*, *Fli1* and *Myb*. These findings also redefine the function of PRC2 accessory proteins, demonstrating that these proteins may fulfill the equivalent role of a core protein in a tissue-specific manner.

## Results

### Mtf2-null mice die in utero from impaired definitive erythropoiesis

Mtf2 is highly expressed in ESCs; however, our in silico analysis of mouse tissue microarrays^25^ determined that *Mtf2* has a more restricted pattern in adults, with higher expression in sites of hematopoiesis. Using intracellular flow cytometry, we analyzed Mtf2 protein abundance in various hematopoietic lineages isolated from bone marrow (BM) and observed that Mtf2 expression is high in long- and short-term HSCs, progenitors (LSK cells) and various stages of erythroblast development (indicated by CD71 and/or Ter119 expression; Supplementary Figure S1a-c). In erythroblasts, Mtf2 expression is modulated during the cell cycle, with highest expression observed during the S and G2/M phases. The dynamic expression of Mtf2 during the cell cycle mirrors that of PRC2 core proteins Suz12 and Ezh2 (Supplementary Figure S1d).

**Fig. 1:**
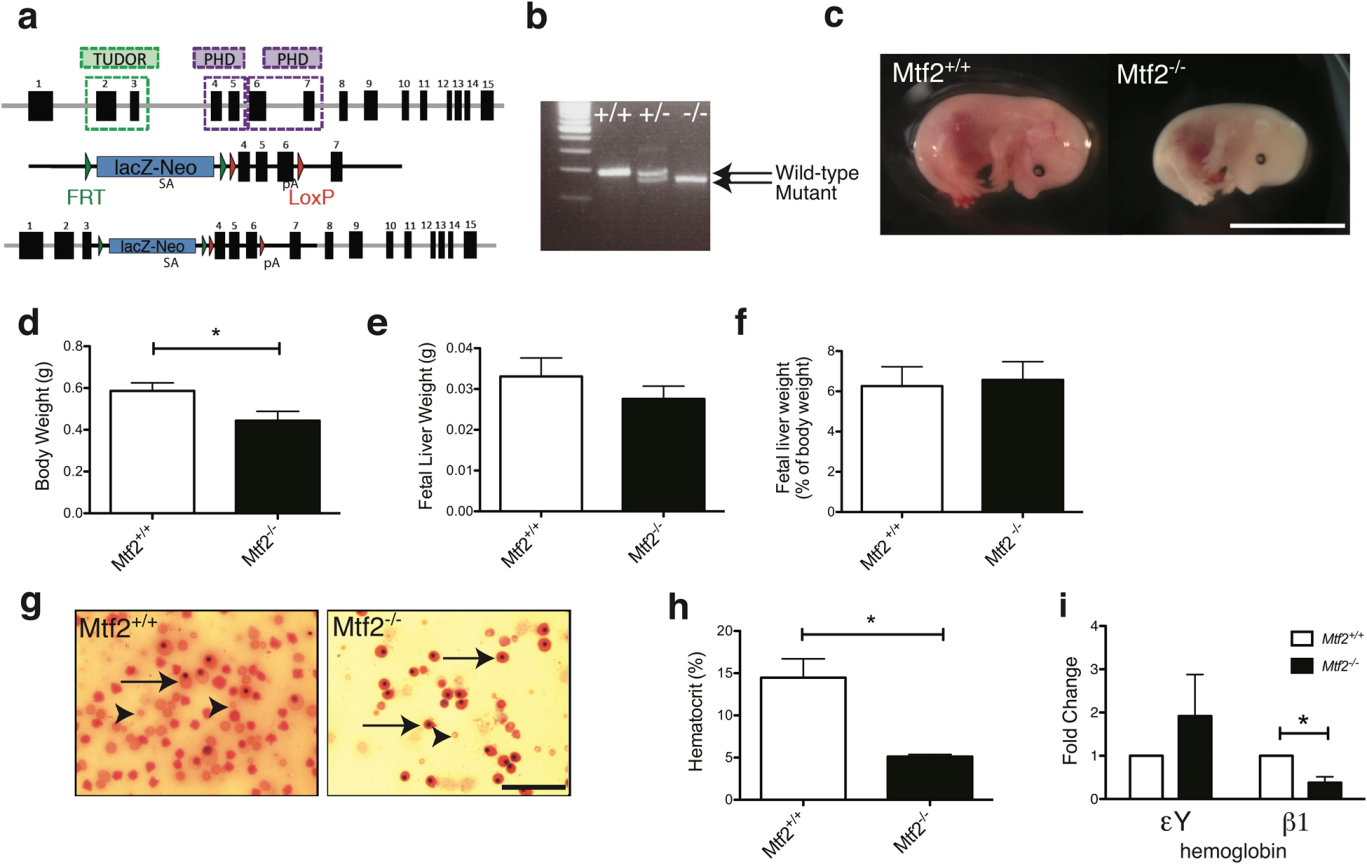
*Mtf2*^*−/−*^ mice die at e15.5 due to severe anemia. **a** Schematic of the gene-targeted ESCs used to create Mtf2 knockout mice. Mtf2 protein domains (Tudor, PHD) are indicated. **b** A PCR-based genotyping strategy was used to identify homozygous mutants. **c** *Mtf2*^*−/*−^ embryos readily display anemia and growth defects, dying by e15.5. **d** The e15.5 *Mtf2*^*−/−*^ embryos are significantly smaller than their wild-type littermates but have **e**,** f** normal fetal liver (FL) weight as a measure of body size. **g** Peripheral blood taken from e15.5 *Mtf2*^*−/−*^ embryos contains fewer cells than wild-type littermates, large nucleated erythroid precursors (arrows) and very few enucleated red blood cells (arrowheads). **h** Null embryos have a lower hematocrit and **i** fail to express adult β1 hemoglobin transcript at the appropriate level, while embryonic (εY) hemoglobin expression was elevated. *P* value was calculated using Student's *t* test. All data are shown as mean ± SEM, *n* = 3, *p < 0.05. See also Supplementary Figure S1

Previous work addressing the role of Mtf2 in vivo was limited to gene trap mutants that displayed variable phenotypes^26,27^. Since gene trap mutations are often hypomorphic^28^ and to maintain strain fidelity, which has also led to variable phenotypes with other polycomb mutants^13,29^, we generated Mtf2-null (*Mtf2*^*−/−*^) mice in the *C57BL/6* background using gene-targeted ESCs to study Mtf2 function in vivo (Fig. 1a, b).

In contrast to the gene trap mutants, no homozygous gene-targeted mice were present at parturition; thus, embryos were analyzed at different developmental stages. *Mtf2*^*−/−*^ embryos die at e15.5, displaying growth defects, hemorrhage and severe anemia (Fig. 1c–e). Embryos also display skeletal alterations, including fusion of vertebrae and ectopic ribs (Supplementary Figure S1e), as observed in the gene trap mutants^26,27^. Based both on the gross pathology of the *Mtf2*^*−/−*^ embryos and the expression pattern of Mtf2 in adult erythroblasts (Supplementary Figure S1c), we further investigated erythroid development in viable *Mtf2*^*−/−*^ e14.5 embryos. At this embryonic stage, the fetal liver (FL) is the central site of hematopoietic development and FL cellularity was significantly reduced in *Mtf2*^*−/−*^ embryos (30.9 ± 1.88 × 10^6^ cells per embryo compared to 64.7 ± 8.95 × 10^6^ cells in wild-type (WT) embryos, *p* = 0.013), albeit FL size as a percentage of body weight was not affected (Fig. 1f). Moreover, peripheral blood smears from *Mtf2*^*−/−*^ embryos showed fewer enucleated mature red blood cells and more nucleated, large primitive erythroblasts compared to *Mtf2*^*+/+*^ controls (Fig. 1g). Hematocrits were also dramatically reduced in *Mtf2*^*−/−*^ embryos (Fig. 1h). In addition, the messenger RNA (mRNA) levels of adult β1 hemoglobin, which is normally expressed by maturing red blood cells at this stage of development, was reduced in *Mtf2*^*−/−*^ embryos while embryonic globin (εY) expression was elevated (Fig. 1i).

**Fig. 2:**
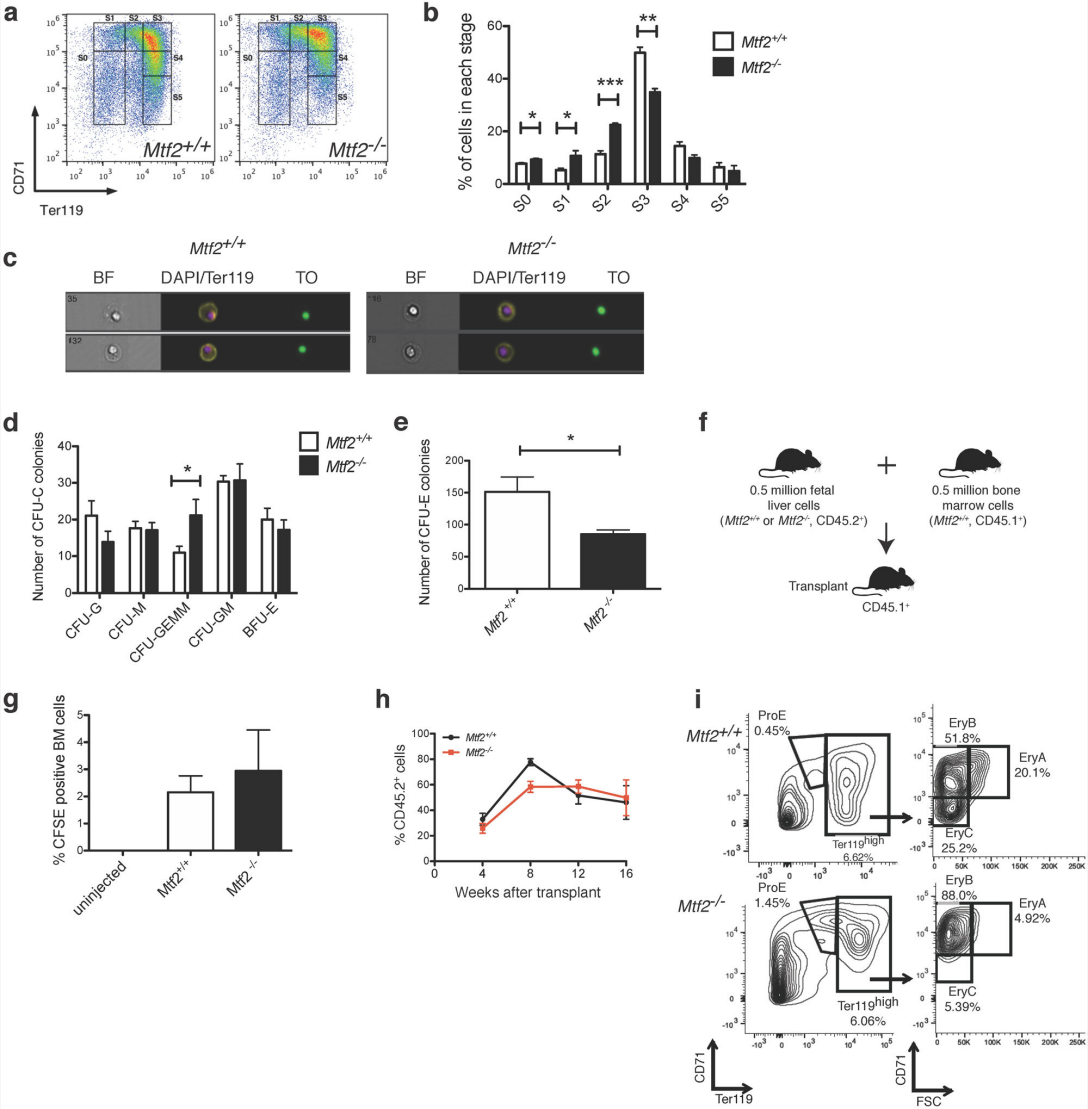
Mtf2 is required for normal erythroid differentiation. **a**,** b** *Mtf2*^*−/−*^ FL cells have increased frequency of pro-erythroblasts (stages S0–S2) and fewer CD71^+^Ter119^+^ erythroblasts (stage S3). **c** Representative pictures from imaging flow cytometry on *Mtf2*^*−/−*^ e14.5 peripheral blood. *Mtf2*^*−/−*^ Ter119^+^ cells have more centrally located nuclei (analyzed by imaging flow cytometry) than their wild-type counterparts (Delta XY centroid values 0.69 ± 0.01 vs. 0.83 ± 0.01, *p* < 0.01). BF brightfield, TO thiazole orange. **d**,** e** FL cells from *Mtf2*^*−/−*^ embryos contain fewer erythroid progenitors (colony-forming unit-erythroid (CFU-E)) but have higher numbers of multipotent clonogenic progenitors (colony-forming unit-granulocyte, erythrocyte, macrophage, megakaryocyte (CFU-GEMM)). CFU-G colony-forming unit-granulocyte, CFU-M colony-forming unit-macrophage, CFU-GM colony-forming unit-granulocyte, macrophage, BFU-E burst-forming unit-erythroid. Data are shown as number of colonies per 2 × 10^5^ FL cells plated, mean ± SEM for *n* = 4 mice, in triplicate. **f **Schematic showing primary repopulation experiments using donor wild-type and *Mtf2*^*−/−*^ e14.5 FL cells. **g** *Mtf2*^*−/−*^ FL cells are able to home to the bone marrow 17 h after injection as well as WT cells. **h** The percentage of CD45.2^+^ donor-derived cells in the peripheral blood of recipient animals is similar when either wild-type or *Mtf2*^*−/−*^ FL donor cells are injected. **i** Erythroid defects observed in *Mtf2*^*−/−*^ FL are cell-intrinsic and recapitulated in recipient mice. A higher frequency of *Mtf2*^*−/−*^ FL donor-derived pro-erythroblasts (ProE) and fewer mature erythroblasts (EryC) were observed compared to WT FL donor cells. *P* value was calculated using Student's *t* test. *p < 0.05, **p < 0.01, ***p < 0.001, *n* = 3. See also Supplementary Figure S2

To discern which stage of erythroid development was blocked in *Mtf2*^*−/−*^ cells, we used the cell surface markers CD71 and Ter119 to track erythroid maturation in the FL^30^. We identified a delay in erythroblast differentiation, with an increased frequency of CD71^+^Ter119^-/lo^*Mtf2*^*−/−*^ cells (erythroid stages S0–S2) and reduced frequency of CD71^+^Ter119^+^ (stage S3) *Mtf2*^*−/−*^ cells (Fig. 2a, b). Despite alterations in cell number between genotypes within FL erythroid sub-populations S2 and S3, cell morphology between genotypes was unaltered, as assessed by imaging flow cytometry (Supplementary Figure S2a-b). Similar to our observations in the FL, we observed increased numbers of pro-erythroblasts (CD71^+^Ter119^lo^, Thiazole Orange^hi^) in the peripheral blood of e14.5 *Mtf2*^*−/−*^ embryos. However, *Mtf2*^*−/−*^ CD71^+^Ter119^hi^Thiazole Orange^hi^ cells that remained in the peripheral blood are more immature than their WT counterparts, as indicated by more centrally located nuclei (Delta XY centroid values of *Mtf2*^*−/−*^ vs. WT: 0.69 ± 0.01 vs. 0.83 ± 0.01, respectively; Fig. 2c). There is also a reduction in the number of reticulocytes and mature red blood cells in *Mtf2*^*−/−*^ e14.5 peripheral blood (0.7% Ter119^-^ DNA^-^ cells vs. 1.89% in WT), corroborating our qualitative observations in blood smears (Fig. 1g). Together, these observations demonstrate that Mtf2 plays a critical role in erythroid maturation.

### Mtf2 is required for erythroid maturation in a cell-intrinsic manner

Since Mtf2 is expressed in various hematopoietic progenitors (Supplementary Figure S1a-b), we assessed the impact of Mtf2 deficiency on the differentiation potential of progenitors using in vitro colony-forming unit (CFU) assays. *Mtf2*^*−/−*^ embryos contained fewer erythroid progenitors (CFU-E) but more multipotent clonogenic progenitors (CFU-granulocyte, erythrocyte, monocyte, megakaryocyte (CFU-GEMM)) than WT embryos, further demonstrating a block in erythroid differentiation potential (Fig. 2d, e). To address the mechanism underlying the observed erythroblast maturation defect, we first determined whether cell cycle and apoptosis was normal in *Mtf2*^*−/−*^ erythroblasts. No increase in Annexin V staining was detected in either *Mtf2*^*−/−*^ CD71^+^Ter119^-/lo^ or CD71^+^Ter119^+^ cells (Supplementary Figure S2c-d). Thus, the reduction in *Mtf2*^*−/−*^mature erythroblasts observed in the FL is not due to enhanced apoptosis. *Mtf2*^*−/−*^ CD71^+^Ter119^lo^ erythroblasts displayed increased cycling with a higher percentage of cells in the S phase and a reduced number of cells in the G_0_/G_1_ phase compared to WT erythroblasts (Supplementary Figure S2e-f). The increased proliferation in these erythroblast progenitors may contribute to their differentiation defect, as erythroid maturation is linked to cell cycle exit^31^. Collectively, these data illustrate that Mtf2 is required for definitive erythropoiesis.

In our constitutive Mtf2 knockout mouse model, Mtf2 is deleted in both the developing hematopoietic cells and the supporting stromal cells of the FL. Therefore, we tested whether the role of Mtf2 in erythroid development is due to the hematopoietic cell microenvironment, as has been previously attributed to the role of Jarid2 in erythroid maturation^12,13^. Using competitive repopulation (Fig. 2f), we observed that *Mtf2*^*−/−*^ and WT FL cells were equally capable of homing to the BM 17 h after injection (Fig. 2g). Additionally, the contribution of donor-derived (CD45.2^+^) cells in the peripheral blood up to 16 weeks following primary transplant was comparable between genotypes (Fig. 2h). Strikingly, analysis of erythroid progenitors demonstrated that *Mtf2*^*−/−*^ donor-derived cells were also defective in erythroid maturation with an accumulation of pro-erythroblasts (ProE) and late basophilic (EryB) erythroblasts (Fig. 2i). This erythroid maturation delay is similar to that observed in the Mtf2-null embryos and therefore not due to a defect in the FL microenvironment. These data demonstrate that Mtf2, unlike Jarid2, plays a cell-intrinsic role in erythroblast maturation that is not limited to fetal development.

### Mtf2 regulates core PRC2 member protein abundance in the hematopoietic system

**Fig. 3:**
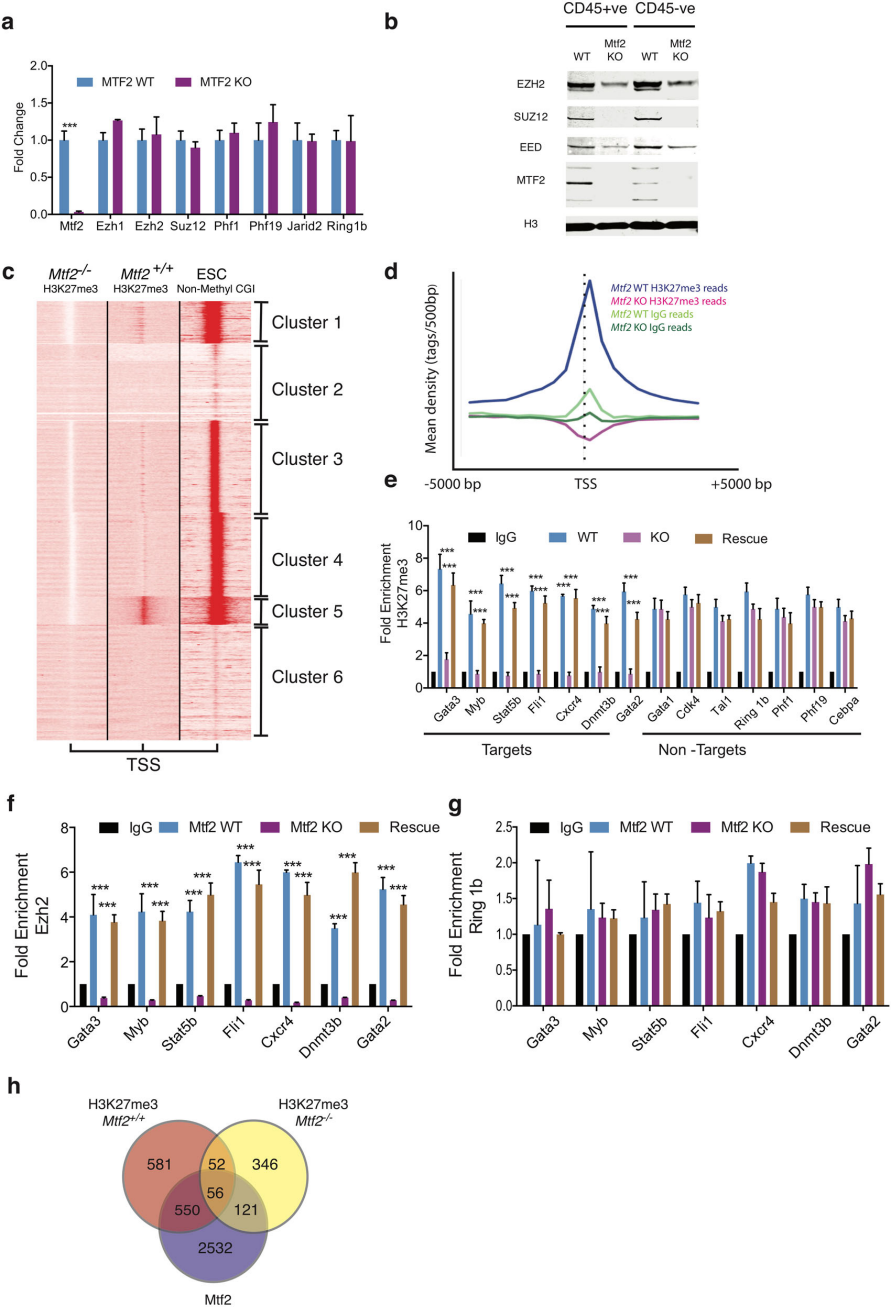
Mtf2 is required for promoter-proximal histone trimethylation of lysine 27 within fetal liver erythroid progenitors. **a** Transcript levels of PRC2 complex members, Suz12, Ezh1/2 and Jarid2, as well as PRC1 component Ring1b are unchanged in *Mtf2*^*−/−*^ CD45^+^ FL cells as determined by RT-qPCR. **b** Core PRC2 proteins Ezh2, Suz12 and Eed are downregulated along with Mtf2 in both CD45^+^ and CD45^-^ FL cells from *Mtf2*^*−/−*^ mice. H3 was used as a protein loading control, and representative images are shown. **c** The *k*-mean clustering identifies patterns of H3K27me3 enrichment in primary CD71^+^ Ter119^+^ erythroblasts. Globally, a loss of enrichment centered around transcriptional start sites (TSS) is observed in CD71^+^ Ter119^+^ cells lacking Mtf2, which correlates with non-methylated CpG islands in ESCs within the same genomic regions. **d** H3K27me3 ChIP-seq density of reads is plotted within 5 kb of the TSS within one cluster of approximately 2400 genes (Cluster 5). In these genes, H3K27me3 binding is specifically reduced immediately around the TSS in Mtf2-null CD71^+^ Ter119^+^ cells. **e** WT and Rescue conditions are compared to KO. Validation of targets and non-targets from H3K27me3 ChIP-seq results performed by ChIP-qPCR. Rescuing Mtf2 expression by overexpressing Mtf2 within Mtf2-deficient CD71^+^ Ter119^+^ cells resulted in increased H3K27me3 binding at positive target loci. **f**,** g** ChIP-qPCR analysis revealed **f** a loss of Ezh2 binding and **g** no changes in Ring 1B binding within TSS regions that showed loss of H3K27me3 marks, when WT and Rescue conditions are compared to KO. **h** Overlap of genes associated with binding sites identified by ChIP-seq. In all, 1131 genes have lost H3K27me3 marks upon loss of Mtf2/PRC2 and 550 of those targets also show Mtf2 binding. See also Supplementary Figures S3-S4. ***P < 0.001

In ESCs, Mtf2 has been identified as a PRC2 accessory protein as it associates with PRC2 core proteins but does not modulate their expression^14,15,26^. Thus, we analyzed the effects of Mtf2 loss on PRC2 expression to determine whether Mtf2 plays a similar role in hematopoietic cells. In *Mtf2*^*−/−*^ erythroblasts, we observed no significant differences in the transcript levels of core PRC2 members such as *Ezh1*, *Ezh2* and *Suz12* or other PRC2 accessory members such as *Jarid2*, *Phf1* and *Phf19* (Fig. 3a). Transcript levels of PRC1 member *Ring 1B* were also unchanged in *Mtf2*^*−/−*^ CD71^+^Ter119^+^ erythroblasts. Surprisingly though, levels of core PRC2 proteins Ezh2, Suz12 and Eed were decreased in *Mtf2*^*−/−*^ sorted e14.5 primary FL cells, including those of hematopoietic origin (CD45^+^) as shown by western blot (Fig. 3b and Supplementary Figure S3a-b). Considering Mtf2 is highly expressed within WT CD71^+^Ter119^+^ erythroblasts (Supplementary Figure S1c) and its expression is synchronous with that of PRC2 core complex members (Supplementary Figure S1d), we next investigated whether restoring the expression of Mtf2 within *Mtf2*^*−/−*^ CD71^+^Ter119^+^ erythroblasts would restore the levels of the PRC2 core complex members. Rescue of Mtf2 expression in *Mtf2*^*−/−*^ FL CD71^+^Ter119^+^ erythroblasts resulted in a concomitant increase in protein levels of core PRC2 complex members and in H3K27me3 levels comparable to those found within the WT CD71^+^Ter119^+^ erythroblasts (Supplementary Figure S3c-g). These data contrast observations of Mtf2 in ESCs, where loss of Mtf2 did not affect PRC2 core members^14^. These data taken together demonstrate that Mtf2 is required for the function of the PRC2 complex in hematopoietic cells, a role more akin to a PRC2 core protein than an accessory protein.

### Mtf2 regulates promoter-proximal H3K27me3 in erythroblasts

To understand the effect of reduced global levels of H3K27me3 at a genomic resolution, we examined the functional consequences of Mtf2 deficiency by chromatin immunoprecipitation sequencing (ChIP-seq). We performed H3K27me3 ChIP-seq in FL-derived (e14.5) cells from two distinct stages of erythropoiesis: CD71^+^Ter119^-/lo^ (stages S1 and S2) pro-erythroblasts and CD71^+^Ter119^+^ (stage S3) erythroblasts. We chose these two fractions of cells since they flank the block in erythroid development we observe in Mtf2-deficient mice (Fig. 2a, b).

Using unsupervised *k*-means clustering to characterize groups of genes with distinct binding patterns of H3K27me3 within WT and *Mtf2*^*−/−*^ CD71^+^Ter119^+^ erythroblasts, we observed that Mtf2-null cells display a genome-wide loss of H3K27me3, specifically around the transcriptional start site (TSS; Fig. 3c, Supplementary Figure S4a). This is even more evident by choosing one cluster (Cluster 5) and plotting read density within 5 kb of the TSS (Fig. 3d), clearly demonstrating a specific loss of H3K27me3 at promoter-proximal regions. Over multiple iterations of clustering, we identified 2404 genes that have strong H3K27me3 binding at the promoter region of WT CD71^+^Ter119^+^ erythroblasts, which is virtually eliminated in CD71^+^Ter119^+^ erythroblasts lacking Mtf2 (Supplementary Table S1). Several targets and non-target regions were independently validated via ChIP-quantitative polymerase chain reaction (qPCR) (Fig. 3e). To investigate the state of other polycomb complexes at these sites, we performed Ezh2 and Ring 1B ChIP on WT and Mtf2 knockout (KO) FL CD71^+^Ter119^+^ erythroblasts. While we observed loss of Ezh2 binding (Fig. 3f) within TSS regions that showed loss of H3K27me3 marks, no changes were observed in Ring 1B binding in *Mtf2*^*−/−*^ CD71^+^Ter119^+^ cells at target region of H3K27me3 loss (Fig. 3g).

Mtf2 has recently been shown to recruit the PRC2 to non-methyl CpG islands (CGIs) located at the promoter-proximal regions^32^. To test whether the TSS regions with depleted H3K27me3 signal were associated with promoters having CGIs, we compared our data with Bio-CAP data from mES cells (GSM1064680) and observed 98.5% overlap (*p* < 2.2e^−16^) (Fig. 3c). Furthermore, this group of 2404 genes that show loss of H3K27me3 signal at the promoter region is highly enriched for master regulators of many key biological processes including members of the *Hox*, *Wnt*, *Gata* and *Lhx* families.

Since Mtf2 recruits PRC2 to chromatin, we examined Mtf2–chromatin interactions by performing Mtf2 ChIP-seq in our two erythroid subgroups. Over 90% of genes harboring H3K27me3 marks in WT cells had reduced H3K27me3 in *Mtf2*^*−/−*^ CD71^+^Ter119^-/lo^ pro-erythroblasts and nearly half (48.9%) of these genes were bound by Mtf2 at this stage of development (Fig. 3h). Interestingly, genes associated with Mtf2 binding peaks in CD71^+^Ter119^-/lo^ pro-erythroblasts or CD71^+^Ter119^+^ erythroblasts show very little overlap with Mtf2 or other PRC2 binding profiles in ESCs^6,14,33^ (Supplementary Figure S4b-e), demonstrating cell type-specific interactions of Mtf2 with chromatin. Further dissection of the Mtf2 ChIP-seq data from the CD71^+^Ter119^+^ erythroblasts revealed that only 22.4% of the Mtf2 peaks were bound to the promoter-proximal regions, while 72.6% of the peaks were found on the gene body (exon and intron) or intergenic regions (Supplementary Table S2). These results are consistent with recent findings that demonstrate Mtf2 has high affinity towards chromatin layered with H3K36me3 marks^32^. Furthermore, the distribution of the Mtf2 peaks were also found to be consistent with that of Ezh2 and Ezh1 as reported previously within primary erythroid cells^34^. Recognizing that ChIP-seq only provides a snapshot of transcription factor binding, genes that lost H3K27me3 in Mtf2-null cells were classified as “Mtf2-PRC2 targets”, although not necessarily direct Mtf2 targets, and the data were used as a first step in mapping the molecular mechanisms underlying the erythroid defects observed in Mtf2-null mice.

### Mtf2 regulates Wnt-dependent erythroid maturation

To investigate the role of Mtf2 in the transcriptional regulation of erythropoiesis, we used RNA-seq profiling of FL erythroid progenitors from the same stages outlined for our ChIP-seq studies. Differential gene expression analysis revealed that 751 genes were significantly misregulated (*p* < 0.05) in *Mtf2*^*−/−*^ pro-erythroblasts, while 2999 genes were misregulated (*p* < 0.05) in *Mtf2*^*−/−*^ CD71^+^Ter119^+^ erythroblasts compared to WT. Within the sets of differentially expressed genes, 92.9 and 98% of them were upregulated in the *Mtf2*^*−/−*^ pro-erythroblasts and erythroblasts, respectively. These results are consistent with the role of Mtf2 as a transcriptional repressor.

**Fig. 4:**
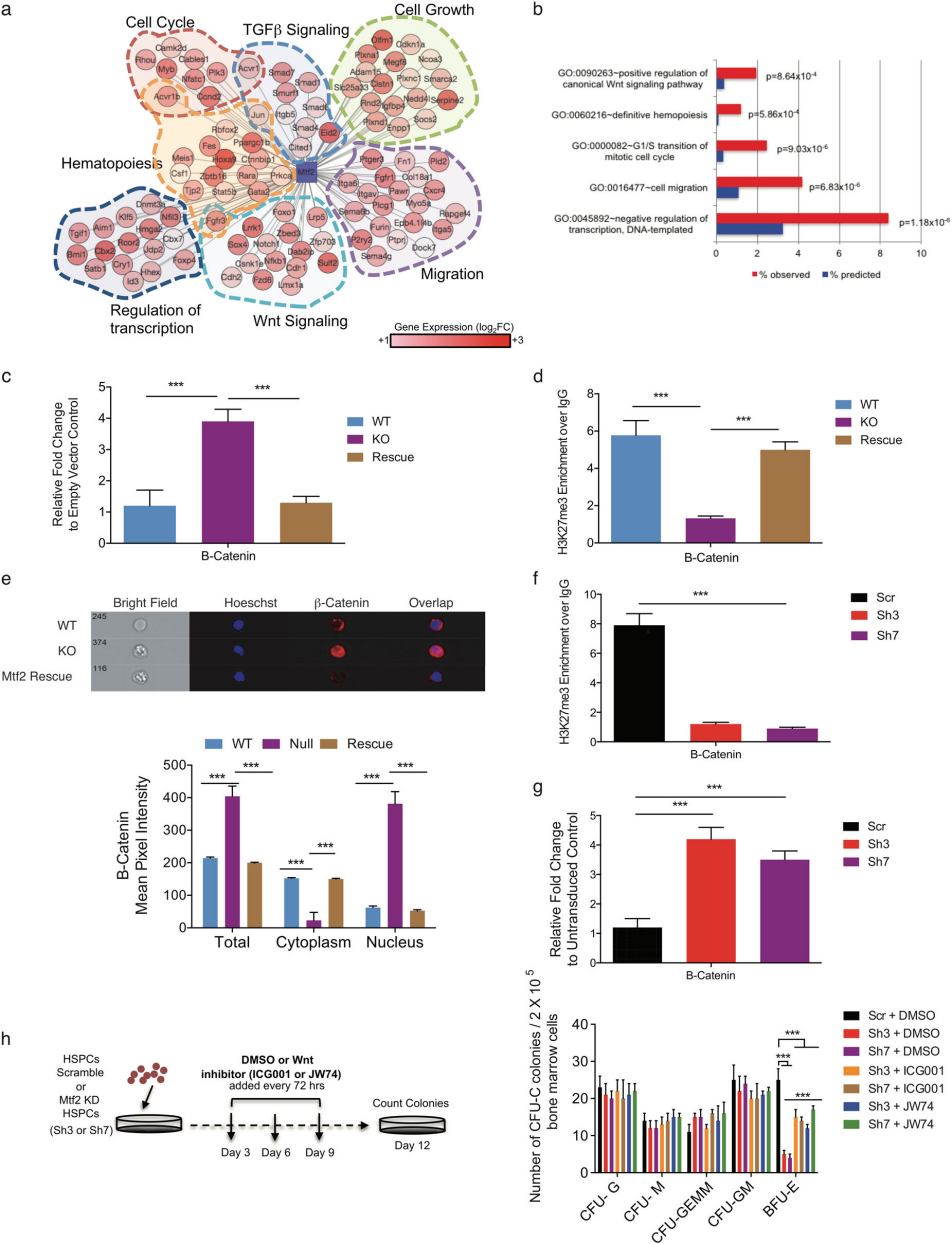
Mtf2 regulates Wnt-dependent erythroid maturation. **a**,** b** An erythroid-specific GRN was drafted using RNA-seq data from WT and Mtf2-null mouse CD71^+^ Ter119^+^ erythroblasts and integrating genes that have lost H3K27me3 upon loss of Mtf2. Node color represents change in gene expression. **b** GO analysis revealed regulatory pathways involved in canonical Wnt signaling, hematopoiesis, cell cycle and transcription are misregulated upon loss of Mtf2. **c**,** d** RT-qPCR and ChIP-qPCR analysis in WT and KO FL HSPCs revealed that **c** β-catenin is overexpressed in *Mtf2*^*−/−*^ FL HSPCs and **d** there is loss of H3K27me3 levels within the promoter region of β-catenin in *Mtf2*^*−/−*^ FL HSPCs. Rescue of Mtf2 levels within *Mtf2*^*−/−*^ FL HSPCs repressed β-catenin mRNA expression, rescued H3K27me3 levels and **e** reduced nuclear localization of β-catenin (detected by imaging flow cytometry, where a mask is created within the nucleus and the mean pixel intensity of β-catenin within the nucleus is compared to that within the entire cell (nucleus+cytoplasm). Knockdown of Mtf2 using two independent shRNAs (Sh3 and Sh7) within adult BM HSPCs led to **f** loss of H3K27me3 levels within the promoter region of β-catenin and **g** increased mRNA expression of β-catenin. **h** Experimental schematic of CFU-C assay, where scramble transduced HSPCs and Mtf2-deficient HSPCs (transduced with two independent shRNAs against Mtf2) were treated with DMSO or Wnt inhibitors (either ICG001 or JW74), respectively, and where all individual treatments of Sh3 and Sh7 KD cells are compared to all individual treatments of Scr transduced cells. Wnt inhibition via ICG001 or JW74 small-molecule treatment in Mtf2-deficient HSPCs gave rise to significantly more BFU-E colonies. ***P < 0.001, *n* = 3. See also Supplementary Figures S5 and S6

To model the effects of Mtf2 on erythroblast maturation, RNA-seq, Mtf2 ChIP-seq and H3K27me3 ChIP-seq data from CD71^+^ Ter119^+^ cells were integrated to draft an erythroid-specific gene regulatory network (GRN). Considering the majority (72.6%) of the Mtf2 ChIP-seq peaks were found within the gene body and intergenic regions (Supplementary Table S2), we decided to focus on the Mtf2 peaks bound to the promoter-proximal regions (22.4%) of genes that showed loss of H3K27me3 levels at the TSS and increased mRNA levels within the *Mtf2*^*−/−*^ CD71^+^Ter119^+^ erythroblasts. These peaks were found to be enriched within regions with non-methyl CGIs^32^ (Supplementary Figure S4f) present at the TSS. This erythroid-specific network consisted of 461 genes controlled by Mtf2-PRC2 (Supplementary Table S3, Supplementary Figure S4f). Gene ontology enrichment analysis revealed this network consists of genes with roles in transcriptional regulation, hematopoietic development, migration, cell cycle and Wnt signaling (Fig. 4a, b).

To test the GRN-based prediction that Wnt signaling is dysregulated in *Mtf2*^*−/−*^ FL hematopoietic stem and progenitor cells (HSPCs), we performed reverse transcription (RT)-qPCR of β-catenin in WT and Mtf2 KO FL HSPCs and observed that β-catenin expression was indeed de-repressed in *Mtf2*^*−/−*^ FL HSPCs relative to WT cells. Moreover, ChIP-qPCR demonstrated decreased H3K27me3 at the β-catenin promoter in *Mtf2*^*−/−*^ HSPCs. Rescue of Mtf2 expression in *Mtf2*^*−/−*^ FL HSPCs restored H3K27me3 at the β-catenin promoter and β-catenin gene repression (Fig. 4c, d). Also consistent with our GRN analysis, which predicted increased Wnt signaling, *Mtf2*^*−/−*^ HSPCs showed increased nuclear localization and decreased cytoplasmic localization of the β-catenin protein as observed by imaging flow cytometry and cytoplasmic β-catenin was restored in Mtf2-rescued cells (Fig. 4e).

To determine the role of Wnt signaling on differentiation potential of adult BM progenitor cells deficient in Mtf2, we used two independent Mtf2 small hairpin RNAs (shRNAs) to knock down *Mtf2* in adult mouse BM HSPCs. As observed in the *Mtf2*^*−/−*^ FL HSPCs, Mtf2 KD BM HSPCs showed increased nuclear localization of β-catenin (Supplementary Figure S5a). Furthermore, knockdown of Mtf2 within adult BM-derived HSPCs also resulted in loss of H3K27me3 mark at the promoter region of β-catenin and increase in its mRNA levels (Fig. 4f, g). These results taken together demonstrate that Mtf2-PRC2 epigenetically represses β-catenin expression within both adult and FL HSPCs.

We then performed functional colony-forming assays in the presence of two different small-molecule inhibitors of activated β-catenin. Both drugs rescued the activated β-catenin levels within the Mtf2-deficient HSPCs and had little effect on the low activated β-catenin levels found within the WT cells (Supplementary Figure S5b-c). The colony-forming assays revealed that Mtf2 knockdown (KD) BM HSPCs form significantly fewer BFU-E colonies than their scramble control counterparts (Fig. 4h). Interestingly, blocking Wnt signaling in Mtf2 KD HSPCs with either inhibitor specific to the canonical Wnt signaling pathway (ICG001 or JW74) resulted in significantly more BFU-E colonies (Fig. 4h). Although the numbers of BFU-E colonies arising from inhibitor-treated Mtf2 KD HSPCs was still lower than that observed from control cells, the rescue of erythroid differentiation to the BFU-E stage was robust and observed using multiple inhibitors and shRNA clone combinations.

**Fig. 5:**
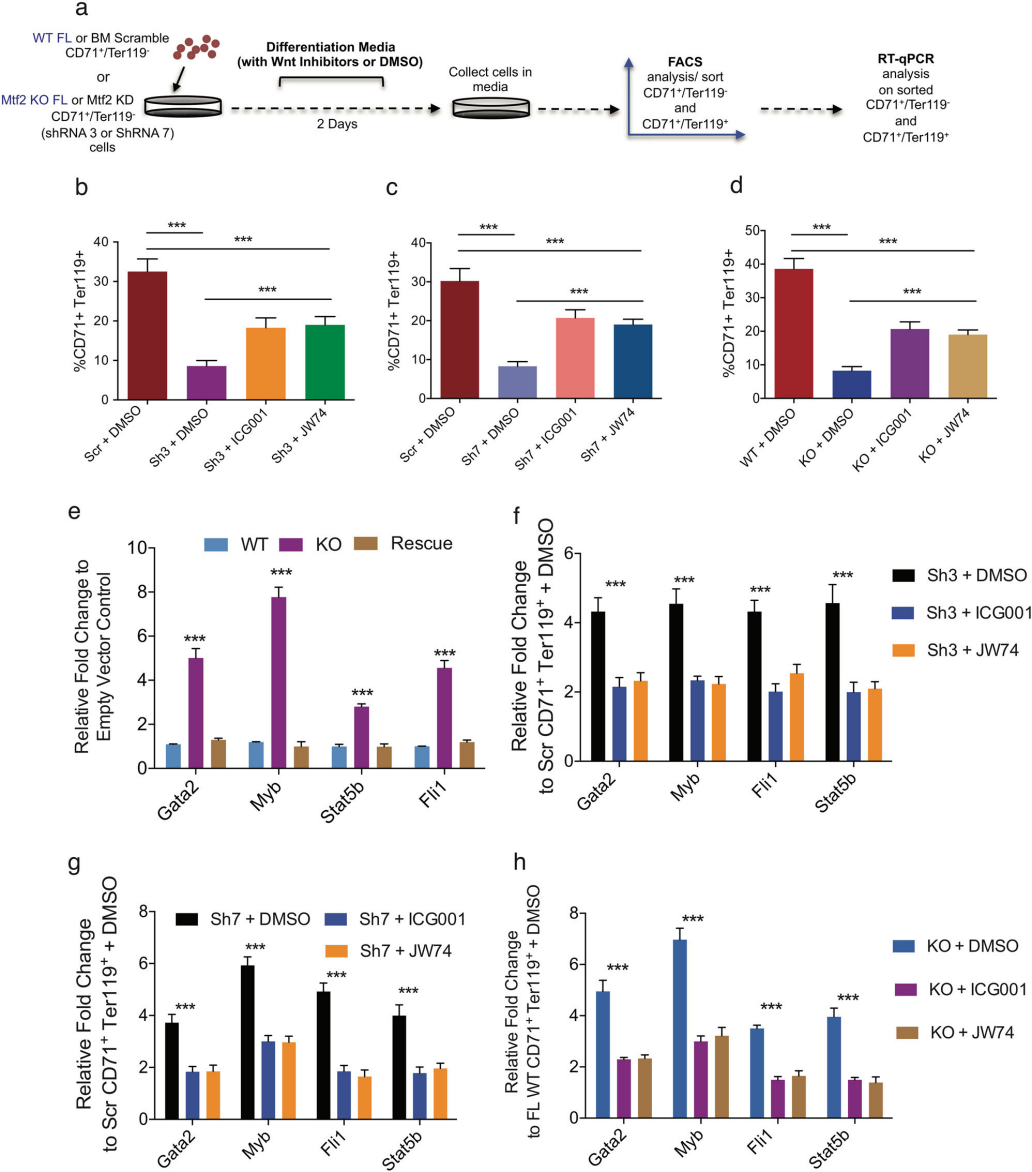
Mtf2-dependent epigenetic regulation of Wnt signaling also affects negative regulators of erythropoiesis. **a** Experimental schematic of ex vivo erythroid differentiation assay using FACS sorted Mtf2-deficient or WT/scramble transduced CD71^+^Ter119^-^ cells. **b**–**d** FACS sorted Mtf2-deficient pro-erythroblasts (CD71^+^Ter119^-^ cells), treated with Wnt inhibitors (ICG001 or JW74) for 2 days in erythroid differentiation culture media, show an increased capacity to differentiate into CD71^+^Ter119^+^ cells. **e**–**h** Mtf2 knockout (**e**,** h**) or knockdown (**f**,** g**) CD71^+^Ter119^+^ cells treated with Wnt inhibitors show inhibition of de-repressed erythroid genes, including *Gata2*, *Myb*, *Fli1* and *Stat5b*. In (**e**), WT and rescue conditions are compared to KO. In (**f**), Sh3 KD cells treated with DMSO are compared to Sh3 KD cells treated with ICG001 and Sh3 KD cells treated with JW74. In (**g**), Sh7 KD cells treated with DMSO are compared to Sh7 KD cells treated with ICG001 and Sh7 KD cells treated with JW74, and in (**h**), KO cells treated with DMSO are compared to KO cells treated with Wnt inhibitors (ICG001 or JW74). ***P < 0.001, *n* = 3

To further demonstrate the impact of Wnt signaling on erythroid maturation, we used an ex vivo erythroid maturation assay^35,36^ in which both FL- and adult BM-derived CD71^+^Ter119^-/lo^ pre-erythroblasts are exposed to differentiation signals, including Epo, and allowed to mature to CD71^+^Ter119^+^ erythroblasts over 2 days (Fig. 5a, Supplementary Figure S6a). Mtf2 KD BM and Mtf2 KO FL pro-erythroblasts are both deficient in their differentiation capacity, as only one third as many cells differentiated to CD71^+^Ter119^+^ erythroblasts, compared with scramble or WT controls (Fig. 5b–d). However, inhibiting Wnt signaling via ICG001 or JW74 treatment during ex vivo differentiation rescued the Mtf2-deficient erythroid phenotype, as demonstrated by the significant increase in CD71^+^Ter119^+^ arising from Mtf2 KD or KO CD71^+^Ter119^-^ pro-erythroblasts following Wnt inhibition (Fig. 5b–d). These data taken together suggest that Mtf2-PRC2 functions in a feed-forward circuit to repress Wnt signaling and allow erythroid differentiation.

### Mtf2-dependent Wnt regulation affects genes critical for erythroid development

**Fig. 6:**
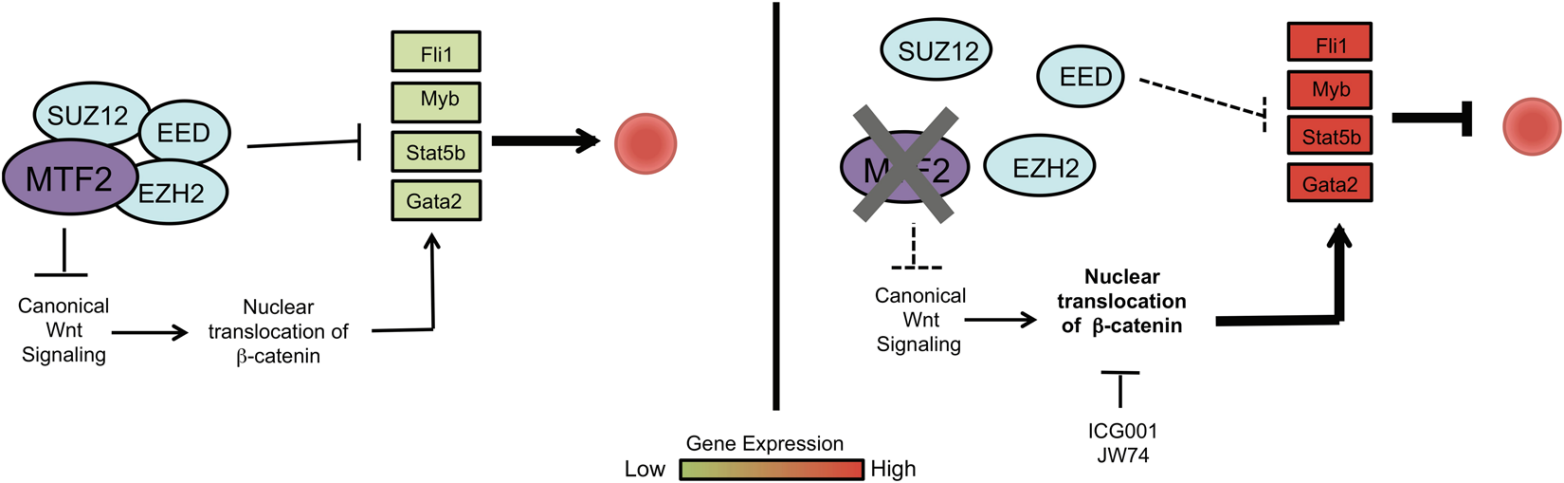
Mechanistic overview of erythroid maintenance via Mtf2 repression of Wnt signaling. In a wild-type erythroblast (left), Mtf2-PRC2 repression of important negative regulators of erythropoiesis and repression of canonical Wnt signaling results in proper erythroid maturation. In the absence of Mtf2 (right), de-repression of erythroid regulators and Wnt signaling results in a block in erythroid differentiation, which can be largely rescued by chemical inhibition of β-catenin

The Mtf2 GRN drafted within FL erythroblasts identified an erythroid-specific module consisting of negative regulators of erythropoiesis, *Gata2, Myb, Stat5b and Fli1*, that are overexpressed within the Mtf2 KO CD71^+^ Ter119^+^ erythroblasts and inhibited upon rescued Mtf2 expression (Fig. 5e). Considering overexpression of these genes has been previously demonstrated to trigger an erythroid differentiation block, Wnt inhibitor-treated adult Mtf2 KD and FL Mtf2 KO CD71^+^Ter119^+^ erythroblasts were analyzed for changes in expression of these critical genes. Interestingly, all the genes within this erythroid-specific module were significantly downregulated upon Wnt inhibition, albeit they still expressed higher than the levels found within WT erythroblasts (Fig. 5f–h). Therefore, using an unbiased systems biology approach, we have identified novel targets of Wnt signaling pathway and a mechanism whereby Mtf2 maintains erythroid differentiation and maturation by repressing the canonical Wnt signaling pathway (Fig. 6) in both adult BM and FL hematopoietic systems.

## Discussion

Using a systems approach, we uncovered a unique and fundamental role for Mtf2 in erythrocyte differentiation, which occurs in part through PRC2-mediated epigenetic repression of canonical Wnt signaling. The cell-intrinsic role of Mtf2 in hematopoiesis and its regulation of core PRC2 complex members re-defines its traditional role as a polycomb accessory protein to an essential core-like PRC2 protein within the hematopoietic system. This novel discovery is in contrast to its role in ESCs^14,15^ and to the role of other PRC2 accessory proteins within the hematopoietic system, such as Jarid2^13,29^. Our findings are also consistent with previously published PRC2 complex purification and proteomics analysis that revealed that Mtf2 within erythroid cells is present at stoichiometric levels within the PRC2 complex^34^. Moreover, at the morphological level, the block in erythroid differentiation and reduced cellularity observed within Mtf2 KO embryos phenocopies that observed within KO embryos of other PRC2 core members^10,11^, further demonstrating that Mtf2 within the hematopoietic system acts as a core complex member. Furthermore, our findings are consistent with PRC1 studies that have shown that both accessory and core proteins play a pivotal role in defining cell type-specific functions in a tissue-specific manner^37–40^.

To elucidate the molecular mechanisms underlying defective erythroid differentiation in Mtf2-deficient mice, we drafted Mtf2 GRNs in erythroblasts and tested the epistatic relationships between Mtf2 and the canonical Wnt signaling pathway. Precise regulation of the canonical Wnt/β-catenin pathway is essential for the development and function of HSCs and defects in the signaling pathway are associated with hematological malignancies^41–43^. Not surprisingly, multiple epigenetic modulators can regulate expression of Wnt pathway constituents^44^. Here, we demonstrate the novel observation that the Mtf2-PRC2 complex transcriptionally represses the expression of β-catenin within both FL and adult mouse HSPCs. Our analysis highlights a specific role for Mtf2-PRC2 in regulating canonical Wnt signaling during erythropoiesis. Inhibition of activated β-catenin by small molecules decreases canonical Wnt signaling which extensively rescued the erythroid maturation defect seen in Mtf2-deficient HSPCs and pro-erythroblasts, as demonstrated by both colony forming and ex vivo maturation assays, respectively. Furthermore, attenuating Wnt signaling via β-catenin inhibition reduced the expression of the negative erythroid regulators, which were de-repressed upon loss of Mtf2. Thus, we have elucidated for the first time a role for PRC2-mediated epigenetic regulation of canonical Wnt signaling in erythroblasts and its downstream effect on genes critical for cell fate decisions.

Tightly regulated epigenetic control of gene expression is critical to coordinate multiple signaling pathways during development. Here, using an unbiased systems biology approach we report a unique role for a PRC2 accessory protein. In a tissue-specific manner, Mtf2 functions similarly to a core PRC2 protein to epigenetically regulate canonical Wnt/β-catenin signaling pathways. Collectively, our data illustrate how Mtf2 functions as an essential PRC2 component in the hematopoietic system, regulating core PRC2 components and modulating PRC2-mediated promoter-proximal H3K27me3 methylation and repression of gene networks that are essential for hematopoietic development and function.

## Materials and methods

### Generation of mice and embryonic analysis

Gene-targeted mouse C57Bl/6 ESCs were obtained through EUCOMM and were aggregated with CD1 blastocysts to form chimeras (https://www.eummcr.org/products/es-cells#targeting). Germline transmission was confirmed using PCR-based genotyping (primers listed in Supplementary Table S4) and all future breeding was done on the C57Bl6/J strain to maintain this background. Peripheral blood analysis on embryos was completed as previously described^45^. Blood smears were stained with Wright-Giemsa. For Mtf2 expression in developing tissues, WT and Mtf2-null mice at various embryonic stages of development were fixed in 4% paraformaldehyde (PFA) and paraffin embedded. Immunofluorescence was performed on 4 µm sections of paraffin-embedded embryos via overnight incubation with antibody against Mtf2 (also known as M96, Genway). Confocal microscopy was performed using a Zeiss Inverted LSM510 microscope. For CFU analysis on FL cells, 2 × 10^5^ cells were isolated and plated in methylcellulose media containing growth factors (Stem Cell Technologies), and colonies were enumerated after 12 days of growth.

### Flow cytometry

For flow cytometric analyses, samples were first subjected to red blood cell lysis and stained with antibodies directed against lineage markers. Mouse peripheral blood, FL or BM was stained with antibodies (from eBiosciences) directed against CD4 (Clone GK1.5), CD8 (53–6.7), CD11b (M1/70), CD71 (R17217), Ter119 (TER119), B220 (RA3-6B2) and Gr1 (RB6-8C5) for lineage analysis. To distinguish donor-derived cells during transplantation, samples were also stained with anti-CD45.2 (104). For mouse stem and progenitor cell analysis, mouse BM and FL cells were first incubated with rat antibodies against CD3 (17A2), CD4, CD5 (53–7.3), CD8, CD11b, Ter119 and B220 and then depleted using Sheep Anti-Rat Dynabeads (LifeTech) (CD11b was excluded in FL analysis). Lineage-depleted samples were then stained with antibodies (from eBiosciences) against cKit (2B8), Sca1 (D7), CD34 (RAM34), CD16/CD32 (93), CD127 (A7R34), CD48 (HM48-1) and CD150 (mShad150). For cell cycle analysis, pregnant dams (e14.5) were injected with 5-bromo-2'-deoxyuridine (BrdU, 1 mg, intraperitoneally) and embryos were harvested 2 h later. Cells were fixed in ice-cold ethanol, treated with 1.5 M HCl for 30 min and then stained with an antibody against BrdU (Millipore) and a fluorescence-conjugated secondary antibody. Cells were stained with propidium iodide (Sigma) to assess cell cycle state. For erythroblast morphology analysis, cells were stained with antibodies against Ter119 and CD71 as above, and with Hoechst (LifeTech) and Thiazole Orange (Sigma) for nucleic acid detection. Cells were analyzed using the ImageStream imaging flow cytometer (Amnis) and classified based on the parameters outlined in ref.^46^. Size measurements were based on area of brightfield images and cells with centrally located nuclei were determined based on delta XY centroid measurements^47^. HSPCs were fixed with 4% PFA, permeabilized with 0.3% Triton and stained for active β-catenin (Millipore) and DRAQ5. The nuclear and cytoplasmic contents of active β-catenin were determined using the “Nuclear Localization” algorithm in the ImageStream software. It is important to note that the signal within the cytoplasm and nucleus is obtained by an unbiased preset-analysis tool within the AMNIS analysis software, where a mask is created within the nucleus and the mean pixel intensity of β-catenin within the nucleus is compared to that within the entire cell (nucleus+cytoplasm). Therefore, the mean pixel intensity takes into account the pixels which comprise the area of the cytoplasm or nucleus in regards to the entire cell (nucleus+cytoplasm).

### Intra-nuclear flow cytometry

BM cells were isolated from C57Bl/6 mice and were first stained with antibodies conjugated to non-tandem dyes. The cells were stained for 15 min at room temperature in phosphate-buffered saline (PBS) containing 2% fetal bovine serum (FBS). Respective isotype controls were used. Once the staining of cells with antibodies conjugated to non-tandem dyes was completed, the cells were washed twice in PBS containing 2% FBS. The cells were then fixed with four antibodies conjugated to non-tandem dyes. The cells were then fixed with 4% PFA for 15 min at room temperature and washed twice with PBS+/+ with 2% FBS and stained with antibodies conjugated to tandem dyes for 15 min at room temperature. Once the fixed cells were stained with antibodies conjugated to tandem dyes, the cells were again washed twice with PBS+/+ with 2% FBS and subsequently permeabilized using 0.3% Triton for 15 min at room temperature. Once permeabilized, the cells were stained with Mtf2 (GenWay, M96 clone) antibody for 30 min on ice. The cells were then washed twice using PBS+/+ with 2% FBS at 4 °C. The cells were then stained with an anti-rabbit secondary antibody. Isotype controls were stained using the above process without staining for Mtf2. Isotype controls were then stained using the same anti-rabbit secondary antibody.

### Lentiviral production of Mtf2 shRNA

The 293T cells were co-transfected with lentiviral plasmids pMD2G, pPAX2 and pGIPZ containing the shRNA (Thermo Scientific) of interest using polyethylenimine (see table below). The supernatant containing the virus was collected 48 and 72 h post transfection. The virus was concentrated through ultracentrifugation and was stored at −80 °C.

**Table:** 

shRNA	Sequence
Mtf2 shRNA Clone 3	TAATGTATGTCATAAGCTC
Mtf2 shRNA Clone 7	TTGGCTTTATGTCCATCCT
Scrambled shRNA	GTTACACGATATGTTATCA

### Lentivirus-mediated Mtf2 knockdown of mouse BM cells

Adult mouse BM was isolated and lineage-depleted to enrich for stem and progenitor cells (Stem Cell Technologies). Cells were maintained in Iscove's modified Dulbecco's media containing bovine serum albumin, insulin and transferrin (Stem Cell Technologies), 100 U/mL penicillin–streptomycin (ThermoFisher), stem cell factor (SCF; 50 ng/mL), thrombopoietin (10 ng/mL), FLT3 (10 ng/mL) and interleukin-6 (IL-6; 10 ng/mL). Growth factors were purchased from Peprotech. On day 1 of infection, cells were incubated with polybrene (6 mg/mL) for 2 h at 37 °C, and then combined with viral supernatants containing either a green fluorescent protein (GFP)-tagged Mtf2 shRNA clone or a scrambled shRNA control (ThermoFisher). Cells were pelleted at 400 × *g* for 20 min, and then maintained at 37 °C. On day 2, infection was repeated. Cells were grown for 3 days using a fed-batch culture system and then sorted according to GFP expression. High GFP cells were fixed in 4% PFA, permeabilized with 0.3% Triton and labeled with antibodies against Mtf2 (Genway), Ezh2 (Cell Signaling), Suz12 (Millipore) or H3K27me3 (Millipore) and stained with the appropriate secondary antibodies. Protein expression was determined by flow cytometry compared with an isotype-only control. Data analysis compared mean fluorescent intensity values using a ratio paired *t* test.

### Colony-forming assays

For CFU assays, 2 × 10^5^ GFP^+^ lentivirus-transduced HSPCs were isolated and plated in MethoCult GF M3434 media containing growth factors (Stem Cell Technologies), and colonies were enumerated after 12 days.

### RNA-seq and ChIP-seq

FL cells from e14.5 *Mtf2*^*+/+*^ and *Mtf2*^*−/−*^ embryos were isolated and CD71^+^Ter119^-/lo^ and CD71^+^Ter119^+^ fractions were sorted directly into lysis buffer by fluorescence-activated cell sorting (FACS). Lineage-negative HSPCs were isolated from FLs of e14.5 *Mtf2*^*+/+*^ and *Mtf2*^*−/−*^ mice using antibodies against lineage markers, as described above. For RNA-seq, RNA was isolated (Arcturus PicoPure Kit, LifeTech) and DNase treated (Qiagen). The quality of RNA was determined using an Agilent 2100 Bioanalyzer. Library preparation was performed using 150 ng high-quality RNA (TruSeq Library Prep Kit, Illumina), and sequenced on a HiSeq 2000 (Illumina). These experiments were done in replicates of two and the replicate data were analyzed using TopHat v1.4.1 and Cuffdiff v1.3.0^48^ to map reads to a reference mouse genome assembly (mm9) and expression differences against the Ensembl release 67 gene model were determined. Significant fold changes were determined using the Benjamini–Hochberg corrected *p*value of 0.05. Raw RNA-seq data are available in Gene Expression Omnibus (GEO; GSE72288). Data were analyzed using the DAVID (the database for annotation, visualization and integrated discovery) bioinformatics tool for functional annotation^49,50^ and Cytoscape with the Enrichment Map plugin for visualization^51,52^. RNA-seq targets were validated by qPCR after RNA was converted to complementary DNA (cDNA) using Superscript II (LifeTech).

For ChIP-seq, sorted cells were crosslinked with 1% formaldehyde for 10 min at room temperature. Samples were sheared using a Covaris sonicator until the DNA reached a final size of 75–700 bp. Then, 10 µg antibody (anti-Mtf2, Genway; anti-H3K27me3, Millipore) was bound to pre-blocked Protein A magnetic beads (Millipore), combined with the sonicated DNA and incubated overnight. After incubation, the beads were collected and the DNA-antibody complexes were eluted at 65 °C. The crosslinks were reversed overnight at 65 °C. Samples were treated with Proteinase K and RNase A and the DNA was purified using phenol–chloroform. A total of 500,000 cells per sample were used for both immunoprecipitation and control (IgG, SantaCruz). While ~ 10 ng of ChIP DNA was obtained from the WT cells, only ~ 6 ng of ChIP DNA was obtained from KO cells, which is consistent with reduced levels of H3K27me3. For sequencing, all the ChIP DNA from 500,000 cells per sample was used for amplification and library preparation (Diagenode Microplex Library Preparation Kit). These experiments were done in replicates of two. DNA was analyzed for quality, quantity and size using an Agilent 2100 Bioanalyzer and digital PCR. Libraries were used for sequencing on a HiSeq 2000 (Illumina). Bowtie v2.2.3^53^ and MACS 1.3.7^54^ were used for alignments and peak calling, respectively. Gene annotations and peak profile analysis were completed using PAVIS^55^ and GREAT^56^. The DAVID bioinformatics tool was used for functional annotation with a Benjamini–Hochberg false discovery rate correction test, unless otherwise indicated^49,50^. Repeated *k*-means clustering analysis of methylation occupancy was completed using seqMINER^57^. ChIP-seq data are available in GEO (GSE72288).

### ChIP-seq validation

The Ezh2 and Ring1b ChIP was performed using clone D2C9 (Cell Signaling Technologies) and clone ab3832 (Abcam) respectively. The ChIP-seq targets were validated by ChIP-qPCR. All qPCR analyses were completed on a Roche Light Cycler 480 using Sybr Green MasterMix (Roche) and 0.1 mM primers. Primer sequences are listed in Supplementary Table  S4.

### H3K27me3 and Bio-CAP data comparison

For comparison of H3K27me3 and Bio-CAP signals, Bio-CAP data from mouse embryonic stem cell V6.5 (GEO sample GSM1064680)^58^ were downloaded as unmapped reads from the Sequence Read Archive (SRA; samples SRR648805 and SRR648806). Reads were mapped to the mm9 genome using bowtie2, BEDtools was used to count H3K27me3 reads from WT and *Mtf2*^−/−^ Ter119^+^ cells and Bio-CAP reads from sample SRR648805 mapping in a range of ±5 Kb from TSSs, and seqMINER was used to perform *k*-means clustering of the three data sets.

### BM transplants and analysis

For homing experiments, 5 × 10^6^ FL cells from *Mtf2*^*−/−*^ or WT e14.5 embryos were isolated and labeled with carboxyfluorescein diacetate succinimidyl ester (CFSE) (5 μM/mL, Molecular Probes) and 5 × 10^6^ washed cells were injected via tail vein into lethally irradiated *B6.SJL-Ptprca Pep3b/BoyJ* (CD45.1^+^) mice (Jackson Labs). BM was harvested 17 h later and assessed for CFSE fluorescence. For primary competitive transplants, 0.5 × 10^6^ million FL cells from either *Mtf2*^*−/−*^ or WT e14.5 embryos (CD45.2^+^) were combined with 0.5 × 10^6^ BM cells from adult CD45.1^+^ mice and injected via tail vein into lethally irradiated CD45.1^+^ mice. Peripheral blood was assessed by flow cytometry at various time points up to 4 months post transplantation.

### Wnt inhibition

The Wnt inhibitors ICG001 (Sigma) and JW74 (Sigma) were dissolved in dimethyl sulfoxide (DMSO). For the CFU, the drugs were added every 72 h directly to the MethoCult GF M3434 media. The Wnt pathway inhibitors, ICG001 and JW74, were added at concentrations of 2 µM and 1.5 µM, respectively. Enumeration of colonies was compared with WT HSPCs transduced with scrambled shRNA and treated with vehicle (DMSO).

### Ex vivo differentiation of erythroblasts

GFP^+^ lentivirus-transduced erythroblasts (CD71^+^/Ter119^-^) were seeded at a density of 1 × 10^6^ cells/mL in StemPro-34 SFM (ThermoFisher) supplemented with l-glutamine (1%; ThermoFisher), Epo (Peprotech; 10 U/mL) and transferrin (Sigma-Aldrich; 1 mg/mL) as previously published^36^. The GFP^+^ cells were cultured for 48 h in the presence and absence of ICG001 (Tocris) and JW74 (Tocris). Half media exchange was performed after 24 h, and after 48 h the cells were collected, stained with CD71 and Ter119 antibodies and analyzed using flow cytometry.

For experiments involving FL cells, FACS sorted CD71^+^Ter119^-^ FL cells were seeded at a density of 0.5 × 10^6^ cells/mL StemPro-34 SFM (ThermoFisher) supplemented with l-glutamine (1%; ThermoFisher), Epo (Peprotech; 10 U/mL) and transferrin (Sigma-Aldrich; 1 mg/mL). The FL cells were cultured for 48 h in the presence and absence of ICG001 (Tocris) and JW74 (Tocris). Half media exchange was performed after 24 h, and after 48 h the cells were collected, stained with CD71 and Ter119 antibodies and analyzed using flow cytometry.

### Western blots

FL cells were stained with CD45 antibody and sorted for the CD45+ve and CD45−ve fractions. The cell nuclear fractionation protocol was performed as previously published^59^. Equal amount of nuclear protein was loaded onto 10-well NuPAGE™ 4–12% Bis-Tris Protein Gels (Life Technologies) alongside 20 µL of Spectra™ Multicolor Broad Range Protein Ladder (Thermo Scientific). The gel was run at 140 V in the XCell SureLock™ Mini-Cell Electrophoresis System (Life Technologies) in 1× MOPS Buffer containing 50 mM Tris-Base (BioShop), 50 mM MOPS, Free Acid (BioBasic), 0.1% (w/v) SDS (Bio-Rad) and 1 mM EDTA (Sigma-Aldrich). The gel was then wet transferred onto methanol activated polyvinylidene difluoride membrane (Millipore) using Mini Trans-Blot® Cell (Bio-Rad) Wet electroblotting system and in 1× Transfer buffer containing 21.5 mM Tris-Base (BioShop), 192 mM Glycine (Sigma-Aldrich) and 20% methanol. After transfer, the membrane was blocked for 1 h using 5% bovine serum albumin (BSA; Wisent Bio products) in Tris-Buffered saline (TBS) with 0.1% Tween-20 (TBS-T; BioBasic). After blocking, the primary antibody cocktail was made using 1:1000 dilutions of Ezh2 (Cell Signalling Technologies, D2C9) and Mtf2 (GenWay, M96) and 1:2000 dilution of Histone H3 (ThermoFisher, 1HH3-3E1) in TBS-T with 5% BSA and the membrane incubated overnight at 4 °C. The membrane was then washed with TBS-T and incubated at room temperature in the secondary antibody cocktail containing 1:10,000 dilution of Invitrogen™ Alexa Fluor 680 Goat anti-rabbit pAb (ThermoFischer Scientific) in TBS-T with 5% BSA for 1 h. The membrane was washed again before visualizing with the Odyssey® classic infra-red imaging system (Li-Cor). Afterwards, the membrane was stripped with Restore™ Western Blot Stripping Buffer (ThermoFischer Scientific) as per the manufacturer’s recommendations, and the western probing was repeated with a new primary antibody cocktail with Anti-SUZ12 (Millipore, 3C1.2) and Anti-Eed (Millipore, AA19) at 1:2000 dilution and the secondary antibody cocktail containing 1:10,000 dilution of Goat anti-Mouse IgG Secondary Antibody DyLight 800 4X PEG (ThermoFishcer Scientific) in addition to the 680 goat anti-rabbit antibody. These experiments were repeated 3 times and every time cells isolated from 3 embryos were combined to generate enough nuclear lysate.

### Production of MTF2 overexpression lentivirus

The 293T cells were co-transfected with second-generation packaging lentiviral plasmids pMD2G, pPAX2 and pLenti vector (abm plasmids) containing MTF2 cDNA or an empty backbone. Supernatant containing the virus was collected 48 and 72 h post transfection.

### Lentiviral-mediated rescue of MTF2 expression within FL HSPCs and CD71^+^Ter119^+^ cells

FACS sorted CD45^+^ cells were lineage depleted using the antibody cocktail mentioned above to obtain FL HSPCs. The FL CD71^+^Ter119^+^ cells were obtained via FACS sorting. The FL HSPCs were cultured in 96-well plates in Stemspan SFEM (Stemcell Technologies) with 50 ng/mL SCF, 50 ng/mL FLT3, 50 ng/mL IL-16, 10 ng/mL IL-13 and 10 ng/mL IL-17 (Peprotech) as previously published^60^. The FL CD71^+^Ter119^+^ cells were cultured in StemPro-34 SFM (ThermoFisher) supplemented with StemPro supplement, 1% penicillin–streptomycin, 1% l-glutamine (200 mM stock), Epo 10 U/mL, SCF 100 ng/mL (Peprotech) and Dexametasone (Sigma) 10^−6^ mol/L. as previously published^36^. The FL cells were transduced at the multiplicity of infection of 10.

### Statistics

All data are presented as mean ± SEM. Data were analyzed using Prism 5.0 (GraphPad Software). Statistical significance of differences was measured by two-tailed Student’s *t-*test. A *p*-value < 0.05 was used as a cut-off to indicate statistical significance.

### Study approval

All animal experiments were conducted with the approval of the University of Ottawa Animal Care Committee in accordance with the Canadian Council on Animal Care Standards and the Province of Ontario’s Animals for Research Act.

## Electronic supplementary material

Supplementary Information(PDF 13773 kb)

Supplementary Table S1(XLS 358 kb)

Supplementary Table S2(XLS 26 kb)

Supplementary Table S3(XLS 54 kb)

Supplementary Table S4(XLSX 47 kb)
