# Supplementary material for: Mtf2-PRC2 control of canonical Wnt signaling is required for definitive erythropoiesis
Source: Cell Discov. 2018 May 1;4:21. doi: 10.1038/s41421-018-0022-5 (PMC5928144; doi:10.1038/s41421-018-0022-5)

Supplementary Information

Mtf2-PRC2 control of canonical Wnt signaling is required for definitive erythropoiesis

Janet L. Manias Rothberg[1,2,3,*], Harinad B. Maganti[1,2,4,*], Hani Jrade[1,2,3], Christopher J. Porter[5], Gareth A. Palidwor[5], Christopher Cafariello[1,2,3], Hannah L. Battaion[1,2,3], Safwat T. Khan[1], Theodore J. Perkins[1,4,5], Robert F. Paulson[6], Caryn Y. Ito[1,3], William L. Stanford[1,2,3,4]

[1]The Sprott Center for Stem Cell Research, Regenerative Medicine Program, Ottawa Hospital Research Institute, Ottawa, ON, Canada K1H 8L6;
[2]Ottawa Institute of Systems Biology, Ottawa, Ontario, Canada;
[3]Department of Cellular and Molecular Medicine, University of Ottawa, Ottawa, Ontario, Canada;
[4]Department of Biochemistry, Microbiology and Immunology, University of Ottawa, Ottawa, Ontario, Canada;
[5]Ottawa Bioinformatics Core Facility, The Sprott Center for Stem Cell Research, Ottawa Hospital Research Institute, Ottawa, ON, Canada K1H 8L6
[6]Department of Veterinary and Biomedical Sciences, Pennsylvania State University, University Park, Pennsylvania 16802, USA

Correspondence: cito@ohri.ca (C.Y.I.); wstanford@ohri.ca (W.L.S.)

[*]These authors contributed equally to this work.

Document contains:

- **Supplementary Figure Legends**
- **Supplementary Figures**
- **Supplementary Tables**

Supplementary Figure Legends

Figure S1. Mtf2 expression in the mouse hematopoietic system. Related to Figure 1. (a) Fixed stem and progenitor cells from mouse bone marrow were analyzed as shown. (b) Mtf2 protein expression is high in $Lineage^{-}Sca1^{+}cKit^{+}$ (LSK) stem and progenitor cells, including LSK $CD48^{-}$ $CD150^{-}$ short term HSCs (STHSC) and LSK $CD48^{-}CD150^{+}$ long term HSCs (LT-HSCs). Solid vertical line indicates level of secondary antibody expression used in staining for intracellular Mtf2. (c) Mtf2 protein expression was analyzed in mature hematopoietic lineages by flow cytometry. Mtf2 is highly expressed in erythroid progenitors but not in T and B cells (n=3). (d) In $CD71^{+}Ter119^{+}$ erythroid progenitors, Mtf2 shows differential expression during different stages of the cell cycle, with highest expression during S phase. Expression of other Polycomb proteins, Suz12 and Ezh2 show a similar expression profile. (e) Skeletal abnormalities present in *Mtf2*$^{-/-}$ embryos in the *C57BL/6* background using gene-targeted ESCs show ectopic ribs and fusion of C1/C2 vertebrae.

Figure S2. Morphology of wild-type and *Mtf2*$^{-/-}$ fetal liver and peripheral blood erythroblasts. Related to Figure 2. (a-b) Fetal liver from e14.5 WT and $Mtf2^{-/-}$ embryos were isolated and stained for markers of erythroblast development (CD71, Ter119). Using imaging flow cytometry, cells from either genotype within the S2 and S3 populations of erythroblasts (A and B, respectively) have similar morphology. BF, brightfield. (c) $Ter119^{-}$ FL cell or (d) $Ter119^{+}$ from $Mtf2^{-/-}$ embryos do not show increased early or late-stage apoptosis, based on Annexin V staining. (e) Representative cell cycle profiles of WT and *Mtf2*$^{-/-}$ $Ter119^{lo}$ and $Ter119^{hi}$ fetal liver erythroblasts were assessed using BrdU and PI staining. In the $Ter119^{lo}$ fraction, there are fewer *Mtf2*$^{-/-}$ cells in G0/G1 and more in S phase. (f) A higher frequency of *Mtf2*$^{-/-}$ $Ter119^{lo}$ pro-erythroblasts are found

in S-phase and fewer in G_o/G1 phase, indicating a defect in cell cycling. Data are shown as mean ±SEM, n=3, *p<0.05.

Figure S3. Mtf2 regulates core PRC2 protein levels and H3K27me3 in erythroblasts. Related to Figure 3. (a-b) Full western blots depicting $CD45^+$ and $CD45^-$ FL cells from *Mtf2*$^{-/-}$ embryos used to create Figure 3B. As shown in Figure 3B, core PRC2 proteins (a) Suz12, Eed and (b) Ezh2 are down-regulated along with Mtf2. H3 was used as a protein loading control. (c-g) Erythroblasts ($CD71^+Ter119^+$) from *Mtf2*$^{-/-}$ e14.5 embryos have reduced protein expression of Mtf2, Suz12, Ezh2, Ezh1 and H3K27me3, compared to erythroid cells from wild-type embryos. Rescuing Mtf2 deficiency by overexpressing Mtf2 in *Mtf2*$^{-/-}$ $CD71^+Ter119^+$ erythroid cells increases levels of other Polycomb proteins back to wild-type levels. Level of total H3K27me3 is also increased in Mtf2 rescue $CD71^+Ter119^+$ cells. (p<0.001, n=3).

Figure S4. Genes associated with Mtf2 binding in erythroid cells show little overlap with Mtf2 and PRC2 targets in ESCs. Related to Figure 3. (a) ChIP-seq tracks show loss of the repressive H3K27me3 marks at the genomic loci of Mtf2 targets: *Gata2*, *Fli1* and *Cxcr4* in *Mtf2*$^{-/-}$ $CD71^+Ter119^+$ erythroid cells. (b) Mtf2 targets determined by ChIP-seq are distinct from Mtf2 targets identified in ESCs. (c-e) Targets of PRC2 members identified in ESCs show little overlap with Mtf2 targets identified in primary $CD71^+Ter119^{-/lo}$ and $CD71^+Ter119^+$ erythroid cells (Mtf2 data (Walker, 2010), Ezh2 & Jarid2 data (Peng,2009), Suz12 & Phf19 data (Hunkapiller, 2013)). (F) The minority of the Mtf2 peaks (22.35%) within *Mtf2*$^{+/+}$ $CD71^+$ $Ter119^+$ cells are found proximal to the TSS region. K-means clustering analysis revealed that genes within the Mtf2-PRC2 GRN

show enrichment of Mtf2 within the promoter proximal regions, along with loss of H3K27me3 and enrichment of non-methyl CGIs within the TSS regions respectively. Peak classification (Supplementary Table S2) revealed that 72.56% of the Mtf2 peaks are present within the intron and intergenic regions of the genome. This pattern is similar to that of Ezh2 (76.68%) and Ezh1 (67.71%) binding profile as previously observed by Xu et al (2015) within human erythroid cells.

Figure S5. Mtf2 epigenetically regulates the expression of the β-catenin. Related to Figure 4. (a) Mtf2-deficient HSPCs express higher levels of β-catenin within their nuclei, compared to control cells infected with scrambled constructs, indicating activation of the Wnt signaling pathway. Protein levels were quantified in single cells using imaging flow cytometry. (b,c) Scramble transduced HSPCs and Mtf2-deficient HSPCs (transduced with two independent shRNAs against Mtf2) were treated with either DMSO or a Wnt inhibitor (either ICG001 or JW74). While no significant effect on nuclear β-catenin levels are observed within scramble transduced HSPCs when treated with ICG001 or JW74, a dramatic loss in nuclear β-catenin levels are observed in Mtf2-deficient cells. **$p<0.01$, ***$p<0.001$, n=3.

Figure S6. Inhibition of the Wnt pathway inhibition increases the ability of Mtf2-deficient erythroblasts to differentiate. Related to Figure 4. (a) Schematic of ex vivo erythroid differentiation assay with Mtf2-deficient (KO or KD transduced with two independent shRNAs against Mtf2) and scramble-transduced or WT proerythroblasts ($CD71^{+}Ter119^{-}$) cells. (b,c) Representative flow cytometry plots reveals Mtf2-deficient proerythroblasts show an increased capacity to differentiate to $CD71^{+}Ter119^{+}$ cells after 2 days *in vitro* when treated with small

molecule inhibitors of Wnt (ICG001 or JW74).

Supplementary Figure S1

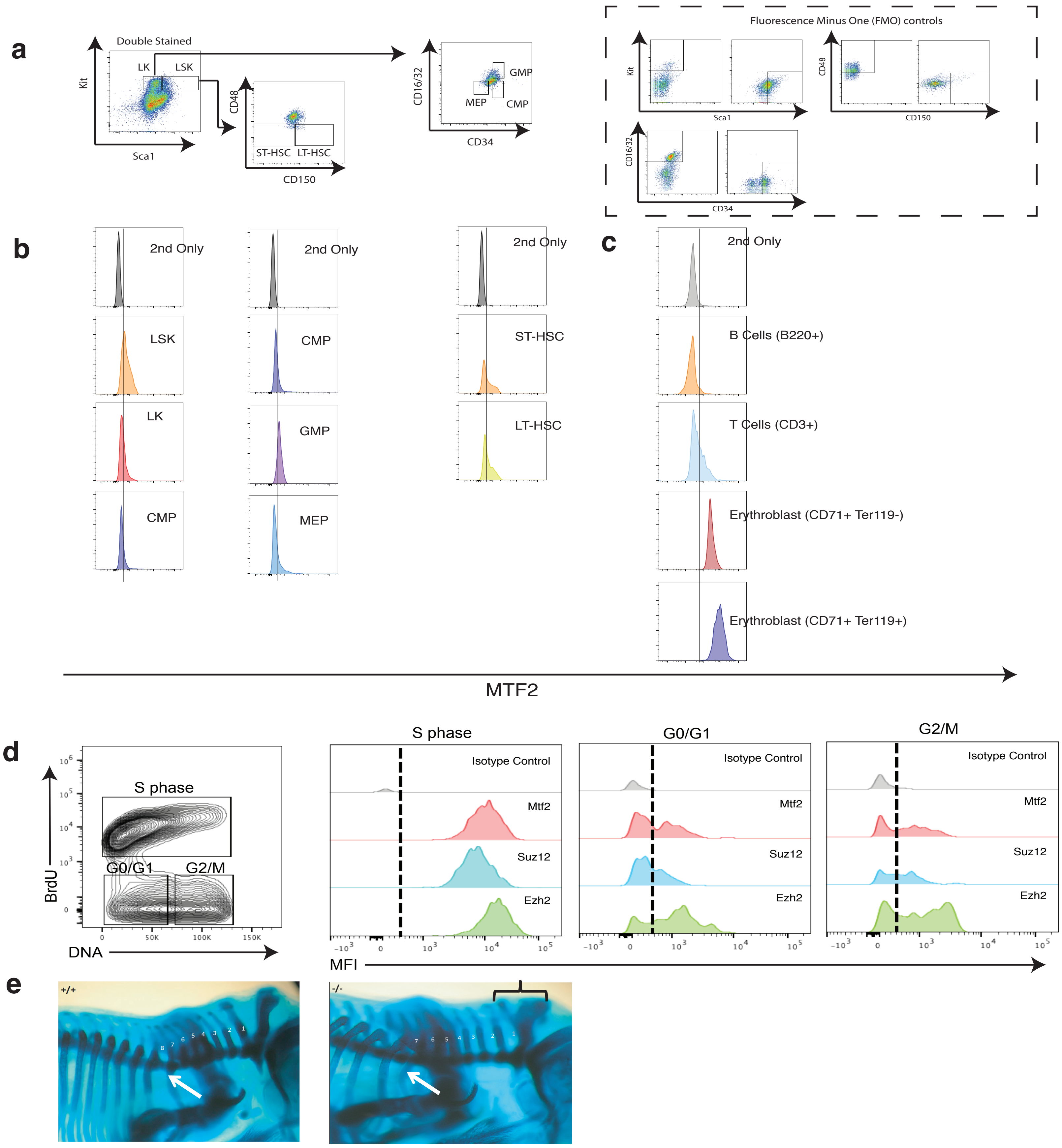

Supplementary Figure S2

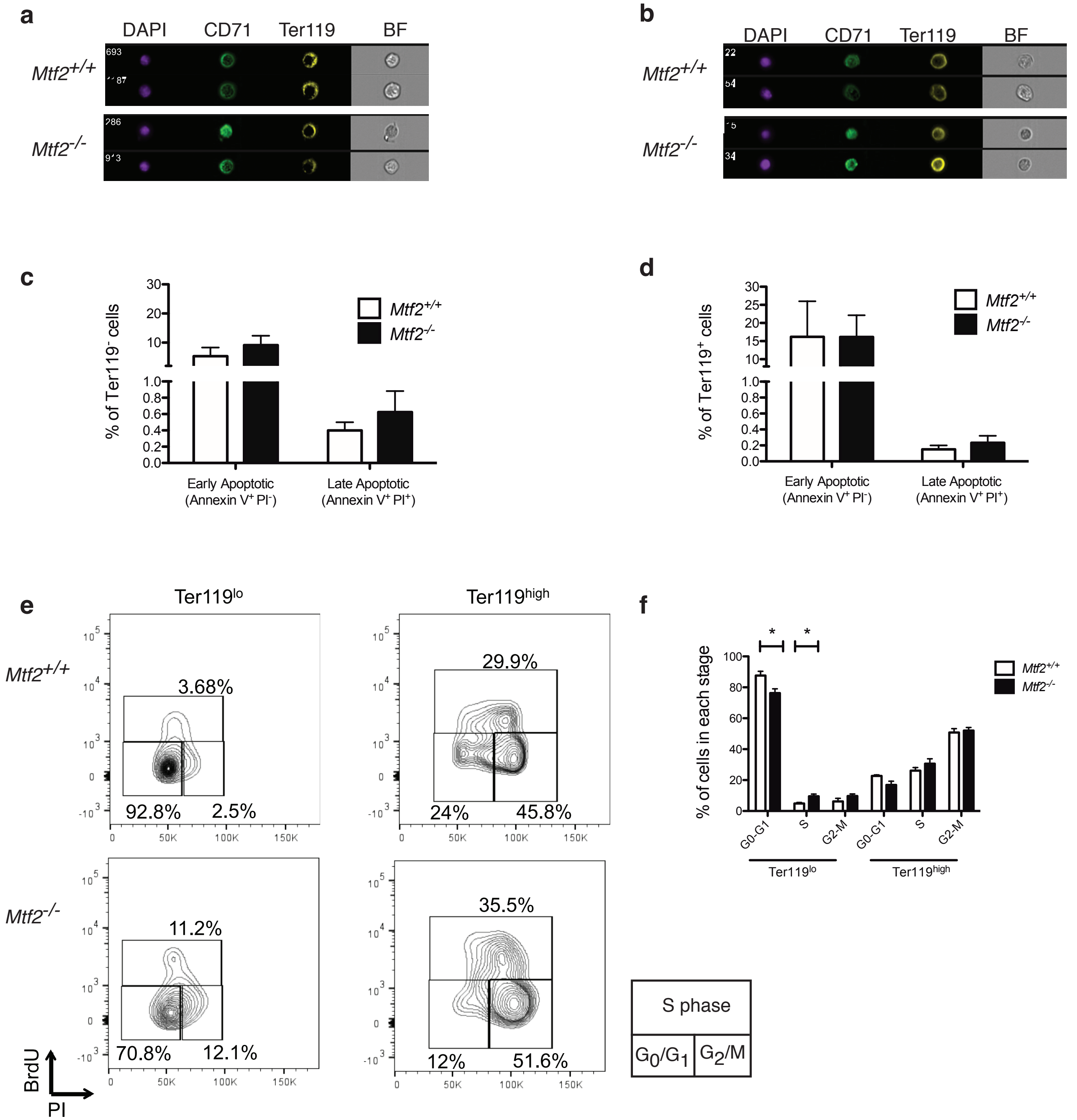

Supplementary Figure S3

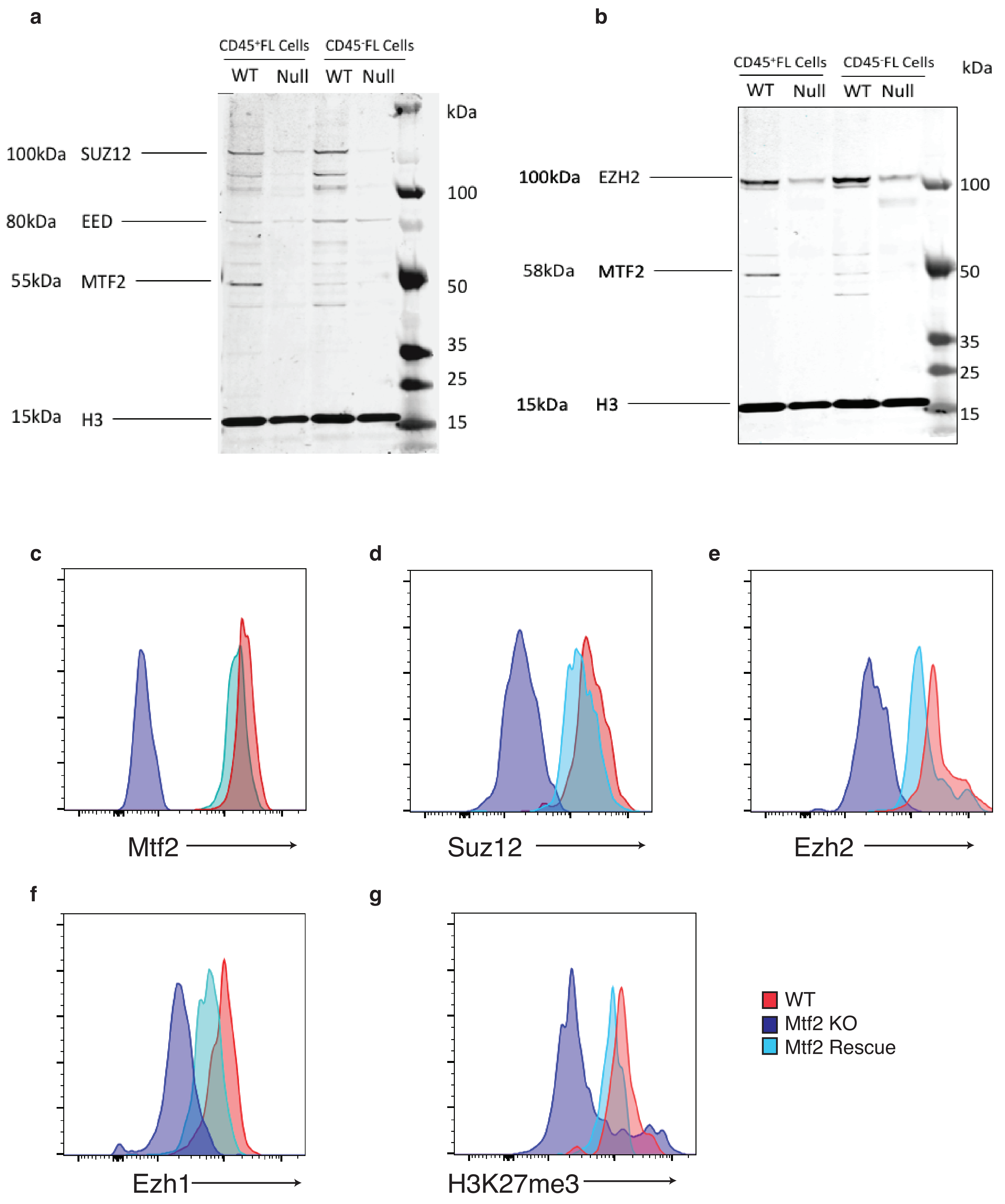

Supplementary Figure S4

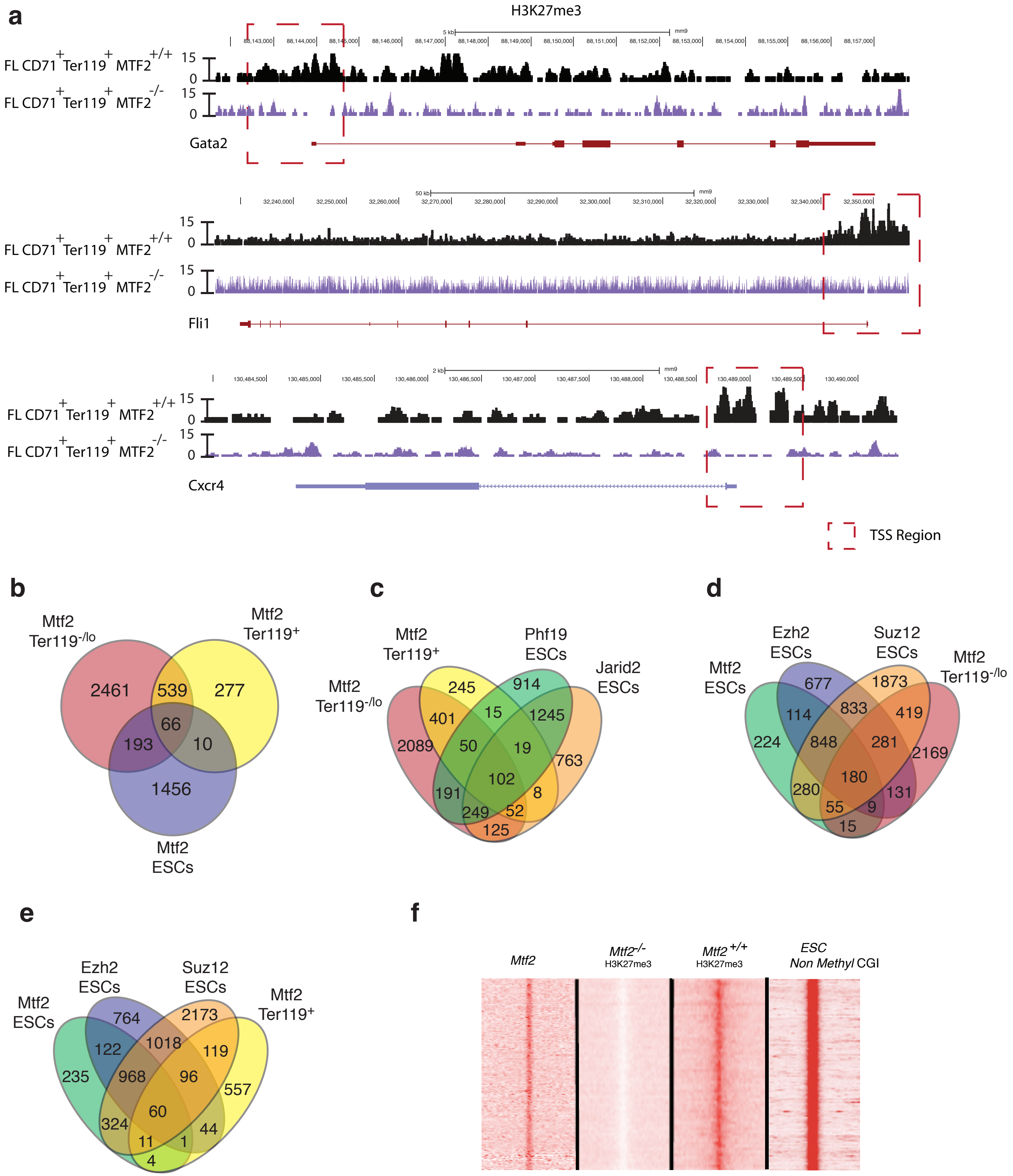

Supplementary Figure S5

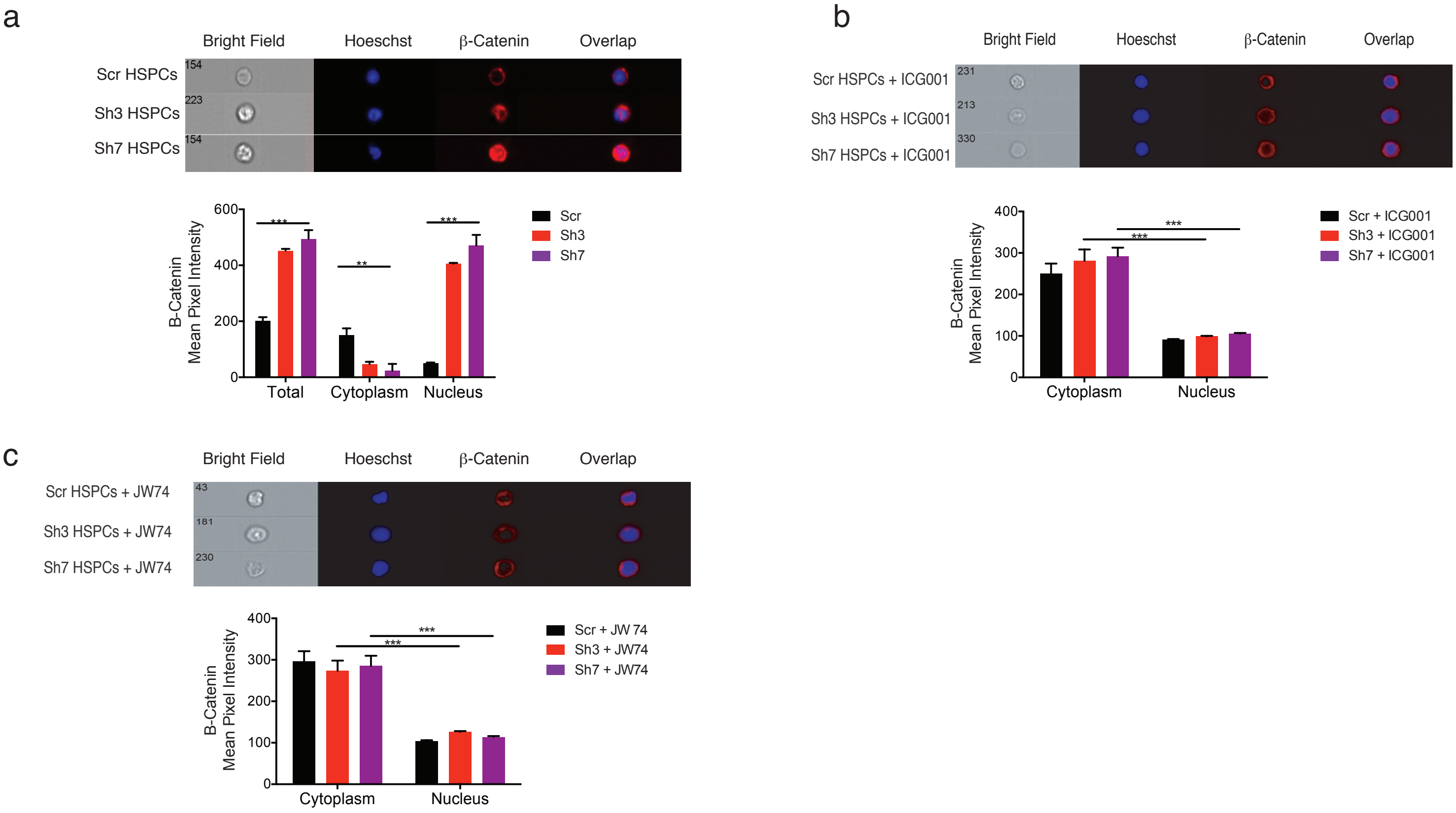

Supplementary Figure S6

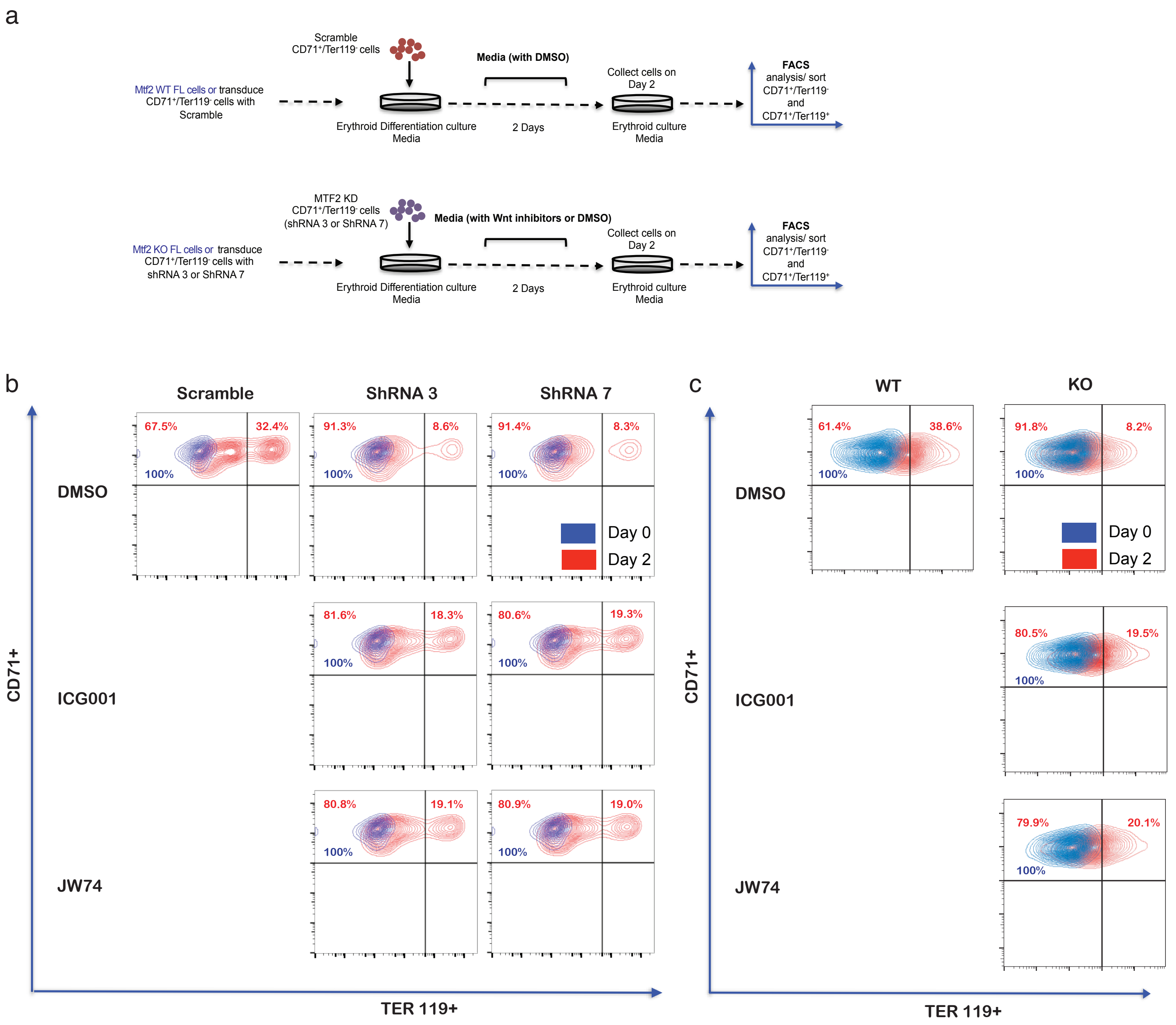

Supplement: Supplementary file 1 — Supplementary Information(PDF 13773 kb) [file 41421_2018_22_MOESM1_ESM.pdf]
